# Classification of Deprivation Indices That Applied to Detect Health Inequality: A Scoping Review

**DOI:** 10.3390/ijerph191610063

**Published:** 2022-08-15

**Authors:** Anastasia Zelenina, Svetlana Shalnova, Sergey Maksimov, Oksana Drapkina

**Affiliations:** National Medical Research Center for Preventive Medicine of the Ministry of Healthcare of the Russian Federation, Petroverigskiy per. 10, 101990 Moscow, Russia

**Keywords:** taxonomy, review, epidemiologic measurements, residence characteristics

## Abstract

Introduction: Many studies around the world are undertaken to establish the association between deprivation and public health indicators. Both separate indicators (e.g., income, education, occupation, public security and social support) and complex models (indices) include several indicators. Deprivation indices are actively used in public health since the mid 1980s. There is currently no clear classification of indices. Methods: In the current review, data related to deprivation indices are combined and analyzed in order to create a taxonomy of indices based on the results obtained. The search was carried out using two bibliographic databases. After conducting a full-text review of the articles and searching and adding relevant articles from the bibliography, and articles that were already known to the authors, sixty studies describing the use of sixty deprivation indices in seventeen countries were included in the narrative synthesis, resulting in development of a taxonomy of indices. When creating the taxonomy, an integrative approach was used that allows integrating new classes and sub-classes in the event that new information appears. Results: In the review, 68% (41/60) of indices were classified as socio-economic, 7% (4/60) of indices as material deprivation, 5% (3/60) of indices as environmental deprivation and 20% (12/60) as multidimensional indices. Conclusions: The data stimulates the use of a competent approach, and will help researchers and public health specialist in resolving conflicts or inconsistencies that arise during the construction and use of indices.

## 1. Introduction

The term “deprivation” was introduced by the American sociologist Stouffer SA in 1949. It denotes the reduction in opportunities to satisfy basic needs—psychophysiological, personal, social [1].

In sociology, there is absolute and relative deprivation. Absolute deprivation describes a condition in which household income falls below a level needed to maintain the basic necessities of life, such as food, security, health services and shelter [2].

In the second half of the XX century as an alternative to the absolute approach to the definition of deprivation, the English sociologist Townsend began to actively develop a relative approach. By introducing the term “relative deprivation”, Townsend established the lack of resources to sustain the diet, lifestyle, activities and amenities that an individual or group are accustomed to, or that are widely encouraged or approved, in the society to which they belong [3].

Within the concept of relative deprivation, Townsend identified material and social deprivation. Social deprivation includes “the roles, relationships, functions, customs, rights and duties of members of society and their subgroups”, and the material one includes “material apparatus (income and unemployment), goods, services, resources, amenities, physical environment and social life”.

Subjective and objective deprivation are also distinguished. Objective deprivation is associated with living conditions, family relationships and behavior in society. It is perceived collectively and recorded in the population census. Subjective deprivation is associated with the attitudes or personal beliefs of the individual and is perceived and assessed individually using a questionnaire during the conduct of special surveys.

An enormous number of studies were conducted around the world to establish the relationship between deprivation and health outcomes for a population, using separate indicators of deprivation, such as income [4,5], education [6,7,8], occupation [9,10,11,12], public security [13] and social support [14].

Although individual deprivation indicators are associated with population health, the causality of this relationship has long been debated. Many mechanisms have been proposed to explain these associations, including limited access to health care, poor nutritional status and poor neighborhoods [15].

It is suggested that individual deprivation indicators are markers of other characteristics that are also associated with health. In this regard, for a more reliable causal inference, many researchers began to create more complex models (indices) to assess the relationship between deprivation and health.

At the moment, many indices have been developed to measure both subjective and objective deprivation: they are actively used in the field of public health.

The indices measuring individual deprivation using the questionnaire as a source of information include: the Evaluation of Deprivation and Inequalities in Health Examination Centres (EPICES) score [16], the deprivation in primary care questionnaire (DiPCare-Q) index [17] and the New Zealand index of socioeconomic deprivation for individuals (NZiDep) index [18].

This review focuses only on indices that measure objective deprivation. This choice is due to the possibility of assessing deprivation at the territorial level.

The following specific objectives were pursued:

To combine and analyze data related to deprivation indices (geographic area level, weighting method, data source, etc.).

To create a list and taxonomy of deprivation indices.

### Practical Aspects of the Development of Deprivation Indexes

Nowadays, there is no clear classification of deprivation indices. Some authors point out only the social and material aspects of deprivation and estimate only the socio-economic factors that have an impact on population health [19,20,21,22,23].

The “classic” indicators for assessing the socio-economic status of the population are income, education and occupation. The most popular socioeconomic deprivation indices among researchers are the Townsend index [24], Carstairs index [25] and Jarman index [26]. These indices were developed in the UK in the late 1980s and are still benchmarks reflecting the essence of material and social deprivation. Index developers pursued different goals. For example, the purpose of the Townsend index was to establish a link between material deprivation and health, so the indicators included in the index reflect only the material aspects of the population’s life (unemployment rate, car ownership and owned housing). The indicators included in the Carstairs index were intended to assess the relationship between socioeconomic inequalities and health outcomes, which implied the use of both material and social characteristics. The Jarman index (UPA8 score) was developed to measure the primary health care needs of populations in different areas and was used by the UK Department of Health to assess the workload of general practitioners in order to provide additional payments to ones who work in areas with high deprivation scores. A similar index (Care Need Index) was also developed in Sweden [27].

The driving force for the creation of indices that aggregate social and material characteristics of the population’s life were the reports of the UK Ministry of Health, so-called “Black Report” [28], “Whitehall” [29] and “Acheson” [30] research, where the link between socioeconomic inequalities and population health was assessed.

Many authors in their studies adapt these indices, taking into account income, national characteristics, traditions, demographic characteristics and the standard of living of the population living in different territories and contexts. For example, every country in the UK (Northern Ireland [31], England [32], Scotland [33] and Wales [34]) releases its own version of the Index of Multiple Deprivation, using a similar methodology but with different deprivation indicators.

The aggregate deprivation index includes some individual deprivation indicators that reflect the deprivation essence of the dynamic systems of society (political, socio-economic, demographic), which undergo changes over time; consequently, it is essential that updated versions are periodically released. Therefore, in the mid 1980s, the majority of the population of Scotland lived in social housing, and this indicator was eliminated from the deprivation index. Over time, the number of social housing decreased by almost 20% and this indicator began to be again characterized by deprivation.

The most common problem with which researchers are faced when creating a deprivation index is choosing an appropriate source of data, using the most accessible one: for instance, census data. When conducting a population census, information is collected from census tracts that reflect the different quantity and quality of the population. The quality of the population is understood as a complex of properties of the population that characterizes its reproductive structure in the system of socio-economic relations, as well as the level of education, qualifications, labor productivity, per capita income, ethnic composition, and migration processes.

This aspect during data analysis can lead to ecological fallacy (aggregation bias, ecological bias). Ecological fallacy occurs when aggregating individual data to the whole society. In his paper on the problem of “ecological bias”, Robinson WS noted that the relationships between two variables that exist at the aggregate level do not always coincide with those that exist between them at the individual level: in the second case, the correlation between them can be much weaker and even have the opposite sign, i.e., a completely different focus compared to the one identified in the first case [35]. In order to reduce the risk of an ecological fallacy, geographical areas with the smallest size of population are preferred because of the population are likely more homogeneous in terms of its socioeconomic characteristics [36]. For this reason, many national statistical offices in different countries create special statistical zones. For example, in France, such territory is called IRIS (Îlot Regroupé pour l’Information Statistique), the smallest administrative unit (1 level) for collecting demographic and socio-economic data. On average, 2000 inhabitants live in this territory. A similar administrative unit in Northern Ireland is the Census Output Area (on average 500 people); in England is the Lower Layer Super Output Areas (1000–3000 people); and in Australia is the Statistical Areas Level 2 (3000–25,000 people). The United States uses a zip code as a reporting unit to measure deprivation at the neighborhood level [37,38].

## 2. Methods

**Figure 1:**
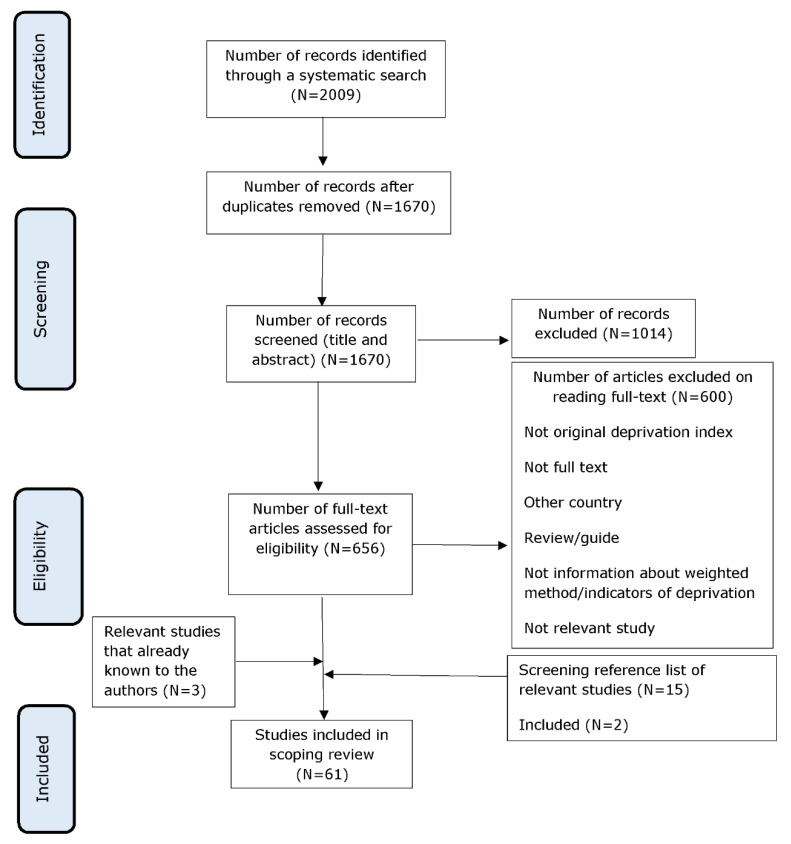
PRISMA-ScR flow diagram of search and study selection process. PRISMA-ScR: Preferred Reporting Items for Systematic Reviews and Meta-analyses for Scoping Reviews.

From 27 January to 24 February 2021, a search was carried out in two bibliographic databases in accordance with the search strategy described in the protocol [39]. The results of the search are presented in a Preferred Reporting Items for Systematic Reviews and Meta-analyses for Scoping Reviews (PRISMA-ScR) flow diagram (Figure 1). The literature search was not restricted by publication year and language. This review considered the deprivation indices developed for the geographic areas of North America, Europe, Australia and New Zealand. There were 2009 records identified in the database. After duplicates were removed, 1670 titles and abstracts were screened. Of these, 656 were included in a full-text review. Finally, after conducting a full-text review and searching and adding relevant papers from the bibliography, and adding papers that were already known to the authors, 60 documents were included in the review. These documents referred to 60 original deprivation indices (see Table S1).

The review included only original indices that are used in public health to measure deprivation and help to find the association between deprivation and health outcomes. We define the original deprivation index as “original deprivation index that include a combination of deprivation indicators that is unique and not repeated in other indices”.

To select only the original indices and exclude the adapted and updated indices, the earliest published papers will be first read at the full-text screening stage. We define an adapted index as “an index (containing a certain (unique) set of deprivation indicators) that is applied outside the country for which it was created”. An updated index is defined as “updated index that is already available, but undergo transformation over time (elimination or addition of deprivation indicators, taking into account social and economic changes in the country)”.

Data were extracted from papers included in the scoping review by two independent reviewers (A.Z. and S.M.). Any disagreements that arose between the reviewers were resolved through discussion or with a third reviewer (S.S.). EndNote X9 (Clarivate Analytics, PA, USA) and the JBI System for the Unified Management, Assessment and Review of Information (JBI SUMARI; JBI, Adelaide, Australia) were used to manage bibliographies and full-text documents (removing, collecting and grouping data).

### Data Extraction and Analysis

To create our taxonomy, we modified the approaches that were used in the studies of Salvador-Carulla et al. [40], Beck et al. [41] and Alexander et al. [42]. They highlighted key topics for their taxonomy by reviewing the published literature. Then, they conducted an expert survey to select the most relevant topics for their classification.

The relevant literature [43,44] and data of the scoping review (see Supplementary Materials) were used to identify four key features of deprivation indices that are used in public health to construct an original deprivation index.

Features of deprivation indices were analyzed in Excel. The extracted features were informed by a scoping review (reported in the protocol).

These became the highest-level category within the taxonomy, denoted as “class”. The data from the literature was also used to create the second level in taxonomy, “subclass”, and the third level, “domain”, applied in the class “type of deprivation”. According to our classification, deprivation indices can have features from several sub-classes (they are not mutually exclusive). When creating the taxonomy, all indicators from which the indices are formed were grouped into domains (groups of different dimensions of deprivation), in accordance with the generality of certain properties of them. Then, the domains were grouped into sub-classes that consolidated in the class “type of deprivation”. Some of the domains in the highest category “type of deprivation” were created, analogous to the domains that were used by researchers in their studies. Some indicators we combined into domains on our own. For instance, we formed the domains that belong to sub-classes “health” and “environmental” using the relevant literature [45,46,47,48].

The following sub-classes are identified in the highest category: “types of deprivation”; “material”; “social”, “health”; and “environmental”. Having studied the literature on this topic, we realized that there is the absence of a clearly delineated border among material, social and environmental deprivation. For example, some researchers believe that education is directly related to income [49,50,51]. Therefore, it can be assumed that education is an indicator of material deprivation. In accordance with Townsend’s concept of relative deprivation, we considered education as a social component of a person’s life in society and adhered to the principle “people with higher education do not always have a high level of income, but they are higher on the social ladder than people without education” and assigned the domain “education” to social deprivation. When creating a deprivation index according to Lalloue et al. [52], the indicators related to old residences were attributed to the domain of “households” and were considered as an indicator of material deprivation, since it was assumed that people did not have enough funds to move to new housing. In the current review, this indicator is classified under the domain “indoor environment”, as it is assumed that materials that are now known to be hazardous to health were used in the construction, interior and exterior finishing of housing in the past (e.g., asbestos, lead paints and plumbing pipes) [53,54,55,56,57].

We attributed “percentage of total census families that are headed by a single female parent” to the domain of “family structure/demographics”; although, according to the same concept of relative deprivation, this indicator should be attributed to the domain of “income”, due to the lower wages of women than men.

In sub-class “environmental”, all the physical, chemical and biological factors external to a person and all behaviors related to the environment are congregated, but excluding behavior related to the social and culture environment.

Any complex model (index) consists of a certain set of factors (indicators), each of which contributes to the result. Often, some parameters are more important than others are. However, it is difficult to determine how great this significance is without the use of special techniques. For this reason, we included separated weighting methods into a separate class in the taxonomy.

Weighting methods were classified according to a study by Schederecker F. et al. [58] that provides more detailed information on each method, and describes the pros and cons of each one, and revealed preferences. Briefly, normative weights: equal weighing, expert weighing, theory-based weighing; as well as input-based weights: statistical weighting (principal component analysis, regression analysis, etc.). 

## 3. Results

### 3.1. Taxonomy of Deprivation Indices

**Figure 2:**
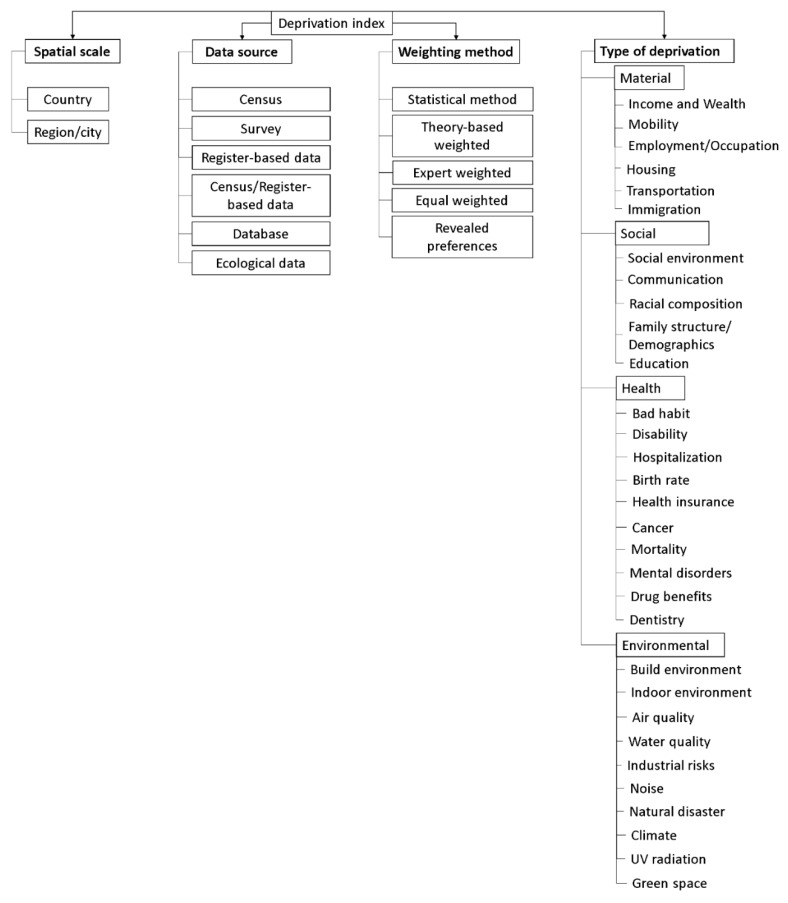
Taxonomy of deprivation indices.

Figure 2 shows the taxonomy of deprivation indices, which consists of four classes: “type of deprivation”, “spatial scale” (which characterizes the scale of the territory where the index is used), “data source” and “weighting method”.

### 3.2. Type of Deprivation

The indices according to class “type of deprivation” are grouped as follows:

- Socio-economic (contains indicators of domains from sub-classes “social” and “material”) [26,59,60,61,62,63,64,65,66,67,68,69,70,71,72,73,74,75,76,77,78,79,80,81,82,83,84,85,86,87,88,89,90,91,92,93,94,95,96,97,98];
- Material (contains indicators of domains from sub-class “material”) [25,99,100,101],
- Environmental deprivation indices (contains only indicators of domains from sub-class “environmental”) [102,103,104];
- Multidimensional indices, which were also divided into indices containing indicators of domains from sub-classes “social”, “material” and “health” [105,106]; “social”, “material” and “environmental” [107,108,109,110]; and indicators of domains from all sub-classes [32,34,111,112,113,114] (see Table S2).

In the review, 68% (41/60) of indices were classified as socio-economic, 7% (4/60) of indices as material deprivation, 5% (3/60) of indices as environmental deprivation, and 20% (12/60) as multidimensional indices.

**Figure 3:**
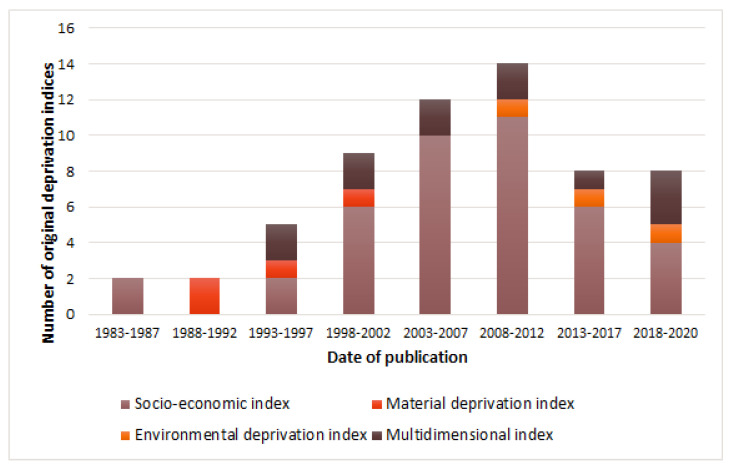
Date of publication of original deprivation indices categorized by type of deprivation.

According to Figure 3, from the late 1980s to the mid 1990s, only two types of indices of socio-economic deprivation and material deprivation were being created. By the early 2000s, there were two new types of indices (multidimensional and environmental deprivation). Yet, we see an increase in socio-economic indices. It can be assumed that until the early 2000s, few basic deprivation indices were mainly used. However, socio-economic and political changes in countries, the development of the theory of social determinants of health (i.e., the emergence of the concepts of “environmental health inequalities” and “social gradient”) and the development of information technologies motivate the creation of new indices.

Indicators belonging to the “employment/occupation” domain were included in 98% (40/41) of socio-economic indices, all indices of material deprivation (4/4) and 91% (11/12) of multidimensional indices. Indicators from “education” were included in 93% (38/41) of socio-economic indices and all multidimensional indices (12/12). All indices of material deprivation, 66% (26/41) of socio-economic indices and 91% (10/12) of multidimensional indices include indicators from the “housing” domain. Indicators from the domain “family structure/demographics” are included in 63% (26/41) of socio-economic indices and 50% (6/12) of multidimensional indices: see Table 1.

Indicators of sub-class “health” are found only in the composition of multidimensional indices, in contrast to ones of sub-class “environmental”, which form both environment deprivation and multidimensional indices. According to the classification, 66% (8/12) of the multidimensional indices include indicators from the “health” subclass, while 75% (6/8) of them include indicators from domain “mortality”; 63% (5/8) of the indices from “disability”; 38% (3/8) of indices from “birth rate”; and 25% (2/8) of indices from “health insurance”, “hospitalization”, “mental disorders” and “cancer”, see Table 1.

Indicators from the sub-class “environmental” are found in 83% (10/12) of multidimensional indices. In general, 46% (7/15) of the indices contain indicators from domain “air quality” (including multidimensional and environmental deprivation indices), 33% (5/15) of the indices include indicators from “industrial risks” and 20% (3/15) of the indices contain indicators from “water quality”, see Table 1.

### 3.3. Spatial Scale

According to the administrative division of the world countries, in the sub-class “region/city”, we included administrative units of all levels (four historical countries in the UK (England, Northern Ireland, Wales and Scotland), regions, provinces, states, districts, cities, etc.).

Most researchers are united in the belief that the most valid indices are those that are developed in separate small areas. This approach allows the inclusion of deprivation indicators that take into account the characteristics of these areas (socio-economic, demographic, etc.).

**Figure 4:**
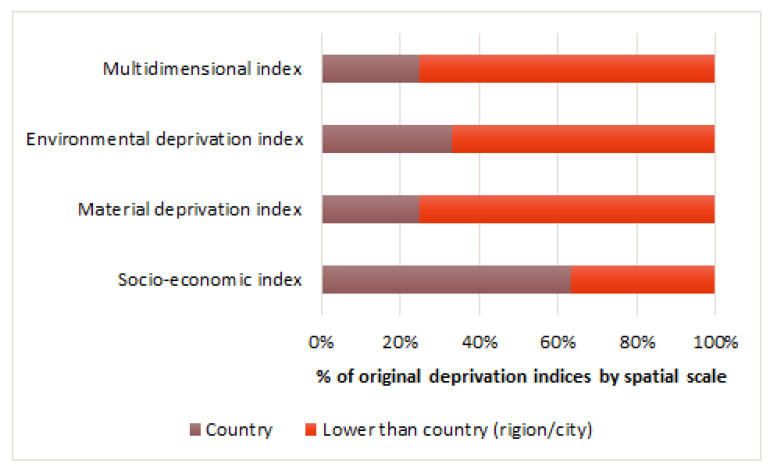
Original deprivation indices grouped by spatial scale.

According to the review, at the country level, 66% (27/41) of socio-economic and 25% (3/12) of multidimensional indices are used. At the regional level—75% (3/4) of material deprivation, 67% (2/3) of environmental deprivation and 75% (9/12) of multidimensional indices (Figure 4).

In the UK and Canada, all indices are developed at the regional level. Similarly, in Spain and Italy most of the indices are developed at the regional level. Denmark, France, the Czech Republic, Hungary, Slovenia, Switzerland, New Zealand, Australia and Sweden developed only national deprivation indices. In the United States, most of the indices are developed at the national level.

### 3.4. Data Source

In class “data source” was included in the following sub-classes: “census”, “survey”, “register-based data”, “census/register-based data”, “database”, “ecological data” (for definitions see Table A1).

When creating 73% (44/60) of indices, the main source of information for extracting deprivation indicators was population census data. In second place is data from survey— 10% (6/60) of indices. A small number of indices were created using mixed data types (sub-class “census/register-based data”) from 7% (4/60) of indices. The California Communities Environmental Health Screening Tool and Child Opportunity Index 2.0 database are data sources for the California Healthy Places Index and Child Opportunity Index, respectively, and were grouped into sub-class “database” from 3% of indices (see Table A2).

### 3.5. Weighting Methods

Statistical weighting was the most popular and most used for more than half of the indices (39/60), particularly for 52% (31/60) of socio-economic indices. Additionally, this weighting method was applied for multidimensional (8/60), environmental deprivation (1/60) and material (1/60) deprivation indices. The equal weighting method was used for creating 23% (14/60) of indices: eight socio-economic indices, three material, one environment deprivation and two multidimensional indices.

**Figure 5:**
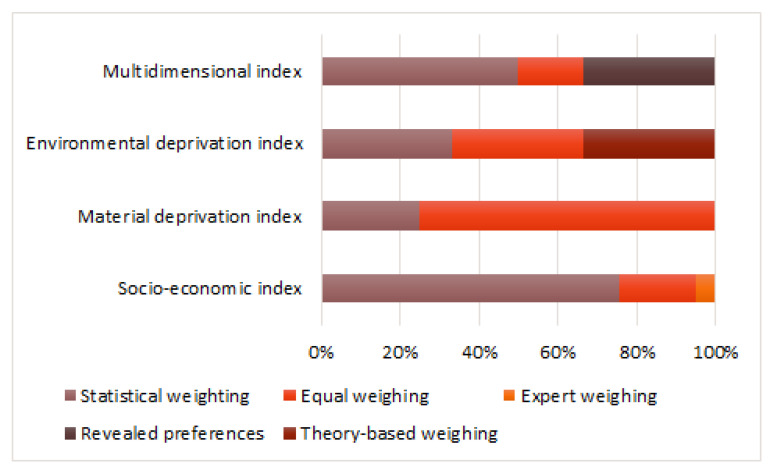
Original deprivation indices grouped by weighting method.

As can be seen from Figure 5, mixed weighting was applied for only two multidimensional indices; theory-based weighting was used for environmental deprivation index; and expert weighting was used only for socio-economic indices (2/41). The pioneer index where this weighing technique was used is the Jarman index, mentioned above.

In the USA, the statistical method (11/15) and the equal weighting method (4/15) were used. Similarly, in Spain, the statistical weighing method was the most commonly used (11/12). In the UK, the most popular was the revealed preferences method (4/11) and the equal weighing method (3/11). In Italy, the method of equal weighting (4/5) was applied.

### 3.6. Using Indices outside of Research

The name of each original deprivation index, along with the name of the first author of the paper, were entered into a Google search engine to search for updated versions of the index and/or deprivation index website and/or additional materials about the index.

Websites/accompanying materials are available for 63% (7/12) of multidimensional indices and 20% (8/41) of socio-economic indices. The indices were created for the UK (9/18), the USA (4/18), Canada (3/18), New Zealand (1/18) and Australia (1/18).

The updated versions have 42% (5/12) multidimensional indexes [115,116,117,118,119], 25% (1/4) material deprivation indexes [120] and only 22% (9/41) socio-economic indexes [52,121,122,123,124,125,126,127,128,129]. No updated versions were found for the environmental indexes (see Table S1).

## 4. Discussion

According to the current review, the USA is the leading country in terms of the number of original deprivation indices (25% of all developed indices (15/60)). Second place is shared by Spain and the UK 20% (12/60) and 18% (11/60) of all developed original indices, respectively. The first deprivation indices (socio-economic) appeared in the early 1980s, and in the early 1990s there were indices of material deprivation. From 1993 to 1997, multidimensional indexes appeared. By the end of the 1990s, there was an upward trend in the creation of new indices, with a predominance of socio-economic indices. For 10 years from 2000 to 2010, the creation of the maximum number of new indices was noted. In the late 2000s, environmental deprivation indices began to be created. Throughout the 37 years of existence of deprivation indices (from 1983 to 2020), socio-economic indices have become the most popular indices for creation: in second place are multidimensional indices; and in third place are environmental indices.

These trends are associated with the use of census data to create indices, which contain mainly socio-economic characteristics and the development of the theory of social determinants of health [130].

Due to this theory, researchers began to go beyond socio-economic approaches in studying health outcomes/evaluating medical care and included indicators associated with components of the environment and health (multidimensional indices) in deprivation indices, and began to create separate indices directly related to deprivation of the environment. One of the obstacles that researchers face in creating such indices is the difficulty of obtaining information on indicators of health, environment and their grouping. As we already know, most researchers use publicly available data to extract deprivation indicators. The most popular of them is the census data (73% (44/60) of indices from the review). This data source has both advantages and disadvantages. The advantage is using a comprehensive research method. A population census commonly refers to the complete count of the persons and housing units found in a country on a fixed date. The disadvantages are the limited set of data (mainly socio-economic indicators), the use of census areas that consists of socio-economically diverse population and the frequency of the population census—once every decade. All of this affect the relevance of the information and is important for creating a valid index. Along with the data of the population census, they also use register-based data that are not publicly available. Examples of register-based data include client information from financial institutions and tax filings. Some indexes include indicators from a mixed data type (both from census data and ad hoc surveys). For example, the European deprivation index [131] includes both indicators from the population census and surveys assessing the quality of life of citizens. Similarly, in the Netherlands, the neighborhood-level deprivation index (the Social Index) was developed using a mixed data source (census data and ad hoc surveys). The survey is conducted using questionnaires that are randomly mailed to residents of neighborhoods [132]. Unfortunately, the indexes mentioned above do not meet the inclusion criteria in our review, therefore, there is not a sub-class ”census/survey” in the taxonomy.

According to the review, only in the UK, Australia, Canada and the USA at the national level, has the collection and grouping of deprivation data concerning not only socio-economic indicators, but also the environmental indicator, been carried out. The advantage of this approach is the interactive monitoring of deprivation indicators, which allows for the updating of these indices as needed. In the United States, the main source of information is the annual survey conducted by the United States Census Bureau. The data collected includes socio-economic and demographic indicators. An interactive data platform (California Communities Environmental Health Screening Tool) is also actively used. The advantage of the platform is to gather data, not only on socio-economic and demographic indicators, but also the environment. This tool allows to receive more complete and operational information both at the state and neighborhoods level [133]. Additionally, the USA Child Opportunity Index 2.0 database was created, which cumulates information from numerous public sources, including the Census Bureau, National Center for Health Statistics (NCHS), Department of Education, the Environmental Protection Agency (EPA) and others.

In Canada, a similar platform was developed—the Canadian Cities Environment and Health Research Consortium (CANUE). CANUE focuses on collating and generating impact indicators in six domains: air pollution, noise, greenness, weather and climate, transportation and neighborhood factors for integration with health databases. This platform contains geospatial data, socio-economic indicators and health data as a whole. The developers suggested that the platform would make it easier for researchers to connect and test a number of their own hypotheses related to the associations between built environment characteristics and health [134].

In Europe, there are no unified databases that combine information on environment and/or socio-economic indicators. For example, to create an index of environmental deprivation in France, researchers used information from several different sources, which, in our opinion, is time-consuming. Moreover, the significance of this index reduces due to the fact that the information received from the sources is not updated simultaneously and annually [104].

Which weighting methods are better to use has to date remained a contentious issue. Until now, scientists have not identified the best method or the “gold standard”. The study by Schederecker F. et al. mentioned above the relationship between territorial deprivation and the overall mortality rate was consistently strong regardless of weighting method. Therefore, it is impossible to give clear recommendations on this issue. As the data from the review show, most researchers prefer the statistical weighting method (52% (31/60) of socio-economic indices), mainly using the principal components method, correlation, factor analysis, and these methods are also used to reduce the number of analyzed variables (deprivation indicators) at the stage of choosing suitable indicators for creating an index. The final decision on the use of the most appropriate deprivation indicators and weighing methods (the so-called validation process is a set of measures to increase validity) is made by the researcher after validating the index. For some authors, the most appropriate way to validate the index is the establishment of a correlation between individual derivational indicators or index and health outcomes (mortality, morbidity, birth rate).

The main limitation of this review is related to the development of a taxonomy based only on deprivation index data developed for New Zealand, Australia, North America and Europe. Therefore, there is a need for an additional review with the inclusion of countries not included in our review, in order to introduce new data into the taxonomy.

## 5. Conclusions

Deprivation indices are widely used in public health research to quantify social and/or environmental inequalities [135,136] and health, or to assess the potentially independent impact of characteristics on health, both at the population and individual level. The influence on health outcomes and the accessibility of health care services, such as life expectancy and survival [137], death in hospital [138], non-communicable disease [139], infectious diseases [140] and trauma [141], are being studied. Furthermore, public health authorities use these indices to make predictions, for example, to identify areas where the need for medical care and the demand for medical services are expected to be the highest, and to target health policies and programs to these areas for the most efficient allocation of health care resources.

The aim of creating a taxonomy was to systematize information related to the methodology for constructing and using deprivation indices in research and practice. To create the taxonomy, an integrative approach was used that allows you to add new classes and sub-classes when new information appears. The terms and definitions introduced during the review are aimed at ensuring uniformity in the taxonomy of deprivation indices and finding a common language among researchers and specialists who develop and use deprivation indices. We also hope that the data from the review will stimulate the use of a competent approach and will help researchers and public health specialists in resolving conflicts or inconsistencies that arise during the construction and use of indices.

## Figures and Tables

**Table 1:** Distribution of indicators of deprivation (domains) depending on the type of index.

Sub-Class/Domain	Socio-Economic Index	Material Deprivation Index	Environmental Deprivation Index	Multidimensional Index	Total
**Income and wealth**	18 (44%)	1 (25%)	-	11 (91%)	30
**Mobility**	3 (7%)	-	-	-	3
**Employment/occupation**	40 (98%)	4 (100%)	-	11 (92%)	55
**Housing**	27 (66%)	4 (100%)	-	11 (92%)	42
**Transportation**	12 (28%)	3 (75%)	-	4 (36%)	19
**Immigration**	7 (17%)	-	-	3 (25%)	10
**Social environment**	1 (2%)	-	-	4 (33%)	5
**Racial composition**	4 (10%)	-	-	3 (25%)	7
**Communication**	5 (12%)	-	-	1 (8%)	6
**Family structure/demographics**	26 (63%)	-	-	6 (50%)	32
**Education**	38 (93%)	-	-	12 (100%)	50
**Health (sub-class)**	-	-	-	8 (66%)	8
**Bad habit**	-	-	-	1 (8%)	1
**Disability**	-	-	-	5 (42%)	5
**Hospitalization**	-	-	-	2 (13%)	2
**Birth rate**	-	-	-	3 (25%)	3
**Health insurance**	-	-	-	2 (13%)	2
**Cancer**	-	-	-	2 (13%)	2
**Mortality**	-	-	-	6 (50%)	6
**Mental disorders**	-	-	-	2 (13%)	2
**Drug benefits**	-	-	-	1 (8%)	1
**Dentistry**	-	-	-	1 (8%)	1
**Environmental (sub-class)**	-	-	3 (100%)	10 (83%)	13
**Build environment**	-	-	-	7 (58%)	7
**Indoor environment**	-	-	-	2 (13%)	2
**Air quality**	-	-	3 (100%)	4 (33%)	7
**Water quality**	-	-	2 (66%)	1 (8%)	3
**Industrial risks**	-	-	3 (100%)	2 (13%)	5
**Noise**	-	-	1 (33%)	1 (8%)	2
**Natural disaster**	-	-		1 (8%)	1
**Climate**	-	-	1 (33%)		1
**UV radiation**	-	-	1 (33%)		1
**Green space**	-	-	1 (33%)	2 (13%)	3
**Total**	41 (100%)	4 (100%)	3 (100%)	12 (100%)	60 (100%)

## Data Availability

The data presented in this study are available in Supplementary Materials.

## Supplementary Materials

The following supporting information can be downloaded at: https://www.mdpi.com/article/10.3390/ijerph191610063/s1. Table S1: Main characteristics of deprivation indices; Table S2: Indicators of deprivation measures. References [25,26,32,34,52,59,60,61,62,63,64,65,66,67,68,69,70,71,72,73,74,75,76,77,78,79,80,81,82,83,84,85,86,87,88,89,90,91,92,93,94,95,96,97,98,99,100,101,102,103,104,105,106,107,108,109,110,111,112,113,114,115,116,117,118,119,120,121,122,123,124,125,126,127,128,129] are cited in the Supplementary Materials.

## Appendix A

**Table A1:** Definitions of sub-classes in class “Data source”.

Sub-Class	Definition
Census	A survey conducted on the full set of observation objects belonging to a given population or universe [142]. Typically, a Census is conducted every 5 or 10 years rather than annually [143].
Survey	An investigation about the characteristics of a given population by means of collecting data from a sample of that population and estimating their characteristics through the systematic use of statistical methodology [142]. A sample survey can be repeated much more often than a Census because it costs less [143].
Register-based data	The data that are in or originate from a register. Register is a database which is updated continuously (often for administrative purposes, such as population registers or building registers) and from which statistics can be extracted/aggregated/computed [143].
Census/Register-based data	The data from a Census and register.
Database	A data file or set of data with relationships expressed among data. Data stored in the database are independent of any particular application [142]. A collection of summary measures about environmental indicators, population characteristics and public health that contribute to human health and well-being [144].
Ecological data	Representation of information about the natural world presented in a structured format suitable for interpretation or processing, that could be reinterpreted for use in a different field of study or context [145].

**Table A2:** Sources of data used to create the deprivation index.

Sub-Class	Total
**Census**	73% (44/60)
**Survey**	10% (6/60)
**Register-based data**	3% (2/60)
**Census/Register-based data**	7% (4/60)
**Database**	3% (2/60)
**Ecological data**	3% (2/60)
