# Supplementary material for: Classification of Deprivation Indices That Applied to Detect Health Inequality: A Scoping Review
Source: Int J Environ Res Public Health. 2022 Aug 15;19(16):10063. doi: 10.3390/ijerph191610063 (PMC9408665; doi:10.3390/ijerph191610063)
Supplement: Supplementary file 1 [file ijerph-19-10063-s001.zip › ijerph-1847394-supplementary.pdf]

Supplementary file

Table S1. Main characteristics of deprivation indices.

#	Name of index/reference (year of publication)	Country (area of a country)	Data source (reporting unit)	Population per Unit (average)	Updates (Author/year of publication)	Weighting method	Website and/or supplementary documentation
**1**	Zadnik et al. (2006) Deprivation Index (DI)[1]	Slovenia	Census (192 municipality)	An average population of 10,304		Bayesian factor analysis	
**2**	Meijer, M. et al. (2013) Danish Deprivation Index (DANDEX) [2]	Denmark	Census (parish)	A median population of 2,545		Principal components analysis (PCA)	
**3**	Benmarhnia et al. (2014) Spatial Environmental Deprivation (SED) Index[3]	France	Ecological data (96 departments)	N/A		Principal components analysis (PCA)	
**4**	Havard et al. (2008) Havard Index[4]	France	Census (190 census blocks)	N/A		Principal components analysis (PCA)	
**5**	Rey et al. (2009) French Deprivation Index (FDep) FDep99 Index[5]	France	1999 Census (30,500 aggregated units for statistical information (IRIS)) / the tax authority's 2001 household income data – Register-based data	A median population of 500	FDep09[6]	Principal components analysis (PCA)	

(Continued)

#	Name of index/reference (year of publication)	Country (area of a country)	Data source (reporting unit)	Population per Unit (average)	Updates (Author/year of publication)	Weighting method	Website and/or supplementary documentation
**6**	Lasbeur et al. (2006) Deprivation Index [7]	France	1999 Census (2,715 aggregated units for statistical information (IRIS))	2,300 people	Lalloue et al. (2013)[8]	Principal components analysis (PCA)	
**7**	Hagedoorn et al. (2016) Municipal Level Deprivation Index[9]	Belgium	2001 Census (Community)	N/A		Equal weighting	
**8**	Splichalova et al. (2007) Index of Socio-Economic Deprivation (SESDI) INDEX1[10]	Czech Republic	2001 Census(52,941 census enumeration districts (EDs))	400 inhabitants		Equal weighting	
**9**	Winkleby et al (2007) Neighborhood-Level Deprivation Index[11]	Sweden	1995 Swedish national database – Register-based data (?) (small area market statistics (SAMS))	1000–2000 people		Principal components analysis (PCA)	

(Continued)

#	Name of index/reference (year of publication)	Country (area of a country)	Data source (reporting unit)	Population per Unit (average)	Updates (Author/year of publication)	Weighting method	Website and/or supplementary documentation
**10**	Ruiz-Ramos et al. (2006) Socio-Economic Synthetic Index [12]	Spain (Seville)	2001 Census (510 census tracts)	N/A		Principal components analysis (PCA)	
**11**	Salcedo et al. (2012) Deprivation Index (DI) [13]	Spain (Barcelona)	2001 Spanish Census of Population and Housing (CPH) (2,978 census tracts)	A median population of 1,085.50		Pena's method (P2 Distance or DP2 method)	
**12**	Dominguez-Berjon et al. (2008) MEDEA Index [14]	Spain (Barcelona, Bilbao, Madrid, Seville and Valencia)	2001Census (census section)(2,358 in Madrid, 1,491 in Barcelona, 598 in Valencia, 510 in Seville and 288 in Bilbao)	An average of 1000 people		Principal components analysis (PCA)	
**13**	Compés Dea et al. (2018) Deprivation Index [15]	Spain (Aragon)	2011 Census (123 Basic Healthcare Areas (BHAs))	From 991 to 34,404 people		Principal components analysis (PCA)	
**14**	Duque et al. (2019) Spanish Deprivation Index (SDI) [16]	Spain	2011 Census (35,917 enumeration districts)	A median population of 19,910 (from 2,163 to 62,260 people)		Principal components analysis (PCA)	

(Continued)

#	Name of index/reference (year of publication)	Country (area of a country)	Data source (reporting unit)	Population per Unit (average)	Updates (Author/year of publication)	Weighting method	Website and/or supplementary documentation
**15**	Colls et al. (2020) Composed Socioeconomic index (CSI) [17]	Spain (Catalonia)	2011 Census (369 basic healthcare areas (BHAs))	A median population of 19,910 (from 2,163 to 62,260 people)		Principal components analysis (PCA)	
**16**	Álvarez-del Arco et al. (2013) Deprivation Index (DI) [18]	Spain (Madrid and Barcelona)	2001 Census (Madrid(2358) Barcelona(1491) census tracts)/ Register-based data	A median population of approximately 1000 inhabitants		Exploratory factor analysis (EFA)	
**17**	Ocaña-Riola et al. (2008) Material Deprivation Index [19]	Spain (Andalusia and Catalonia)	1991Census (3572 census sections 1556 in Andalusia and 2016 in Catalonia)/ Register-based data	N/A		Principal components analysis (PCA)	
**18**	Benach et al. (1999) Deprivation Index [20]	Spain	1991 Census	3,500 inhabitants		Exploratory factor analysis	
**19**	Sánchez-Cantalejo et al. (2008) Deprivation Index [21]	Spain	2001 Population and Housing Census (8,108 municipalities)	N/A		Principal components analysis (PCA)	

(Continued)

#	Name of index/reference (year of publication)	Country (area of a country)	Data source (reporting unit)	Population per Unit (average)	Updates (Author/year of publication)	Weighting method	Website and/or supplementary documentation
**20**	García-Gil et al. (2004) Social Index [22]	Spain (Seville)	1991 Census (32 basic health zones (BHZs), 85 urban neighborhoods)	N/A		Equal weighting	
**21**	Esnaola et al. (2006) Deprivation Index [23]	Spain (the Basque Country)	1996 Census (census section)	An average 1,257 of inhabitants		Principal components analysis (PCA)	
**22**	Panczak et al. (2012) Swiss Neighborhood Index of Socio-Economic Position (Swiss-SEP) [24]	Switzerland	2000 Census	N/A		Principal components analysis (PCA)	
**23**	Juhász et al. (2010) Hungarian Deprivation Index [25]	Hungary	2001 Census (3152 Municipalities (LAU2)) / Register-based (tax) data	Mean: 3,253 people		Principal components analysis (PCA)	
**24**	Caranci et al. (2010) Italian Deprivation Index [26]	Italy	2001 General Census (352,605 census tracts)	An average 169 of inhabitants	Rosano et al.(2020)[27]	Equal weighting	
**25**	Ivaldi et al. (2001) Genoa Deprivation Index [28]	Italy (Genoa)	1991 Census (71 “Unità Urbanisticas” (UUs))	9,557 individuals, min: 1,143, max: 30,981		Equal weighting	
**26**	Cadum et al. (1999) National Deprivation Index [29]	Italy	1991 Census (8,100 municipalities)	N/A		Equal weighting	

(Continued)

#	Name of index/reference (year of publication)	Country (area of a country)	Data source (reporting unit)	Population per Unit (average)	Updates (Author/year of publication)	Weighting method	Website and/or supplementary documentation
**27**	Michelozzi et al. (1998) Deprivation Index [30]	Italy	Census (6,108 census tracts)	An average of 480 inhabitants		Principal components analysis (PCA)	
**28**	Petrelli et al. (2006) Deprivation Index [31]	Italy(Turin)	1991 Census (neighbourhood)	A median population of 41,139 inhabitants		Equal weighting	
**29**	Jordan et al. (2004) Index of Multiple Deprivation 2000 [32]	England	2000 Census (34,400 Lower Super Output Areas (LSOAs))	1,519 people (the mean population size)	IMD04, IMD07, IMD10, IMD15, IMD19 [33]	Revealed preferences	available
**30**	Townsend et al. (1988) Townsend Material Deprivation Score [34]	England	1981 Census (9265 census wards)	N/A		Equal weighting	
**31**	Jarman (1983) Jarman Score [35]	England	1981 Census (postcode sectors(PCSs))	N/A		Expert weighting	available
**32**	Carstairs et al. (1991) Carstairs Index [36]	Scotland	1981 Census (9265 census wards)	N/A		Equal weighting	
**33**	Simpson et al. (1994) Index of Local Conditions (ILC) [37]	England	1991 Census (enumeration districts (EDs))	N/A	Index of Local Deprivation 1998 (ILD)[38]	Equal weighting	available
**34**	Richardson et al. (2010) Multiple Environmental Deprivation Index (MEDIx) [39]	England	Ecological data (10,654 area statistic wards)	5,518 people		Theory-based weighting	available

(Continued)

#	Name of index/reference (year of publication)	Country (area of a country)	Data source (reporting unit)	Population per Unit (average)	Updates (Author/year of publication)	Weighting method	Website and/or supplementary documentation
**35**	Noble et al. (2001) Northern Ireland Multiple Deprivation Measure (NIMDM) [40]	Ireland	1984 Census (electoral ward)	2,000 people	NIMDM05, NIMDM10,NIMDM 17[41]	Revealed preferences	available
**36**	Kelly et al.(1997) Small Area Health Research Unit (SAHRU) Deprivation Index [42]	Ireland	1991 Census (3,409 electoral divisions(EDs))	N/A	SAHRU Index 2006, 2011, 2016[43]	Principal components analysis (PCA)	available
**37**	Haase, Pratschke and Gleeson (2012) All-Island Deprivation Index [44]	Ireland	2011 Census (23,025 small areas (SAs))	N/A		Confirmatory factor analysis(CFA)	available
**38**	Welsh Index of Multiple Deprivation (WIMD) (2000) [45]	England (Welsh)	2000 Census (1,909 Lower-layer Super Output Areas (LSOAs))	1,600 people	WIMD04, WIMD05, WIMD08, WIMD09, WIMD10, WIMD12, WIMD14, WIMD17, WIMD19[46]	Revealed preferences	available
**39**	Scottish Index of Multiple Deprivation (SIMD) (2004) [47]	Scotland	Register-based data (6 976 data zones)	700-800 people	SIMD06, SIMD09, SIMD12, SIMD16, SIMD20[48]	Revealed preferences	available

(Continued)

#	Name of index/reference (year of publication)	Country (area of a country)	Data source (reporting unit)	Population per Unit (average)	Updates (Author/year of publication)	Weighting method	Website and/or supplementary documentation
**40**	Salmond et al. (2012) New Zealand's Deprivation Index (NZDep) [49]	New Zealand	Census (meshblock)	Median population size is approximately 90 persons	NZDep1996, NZDep2001, NZDep2000, NZDep2006, NZDep2013, NZDep 2018[50]	Principal components analysis (PCA)	available
**41**	Ross index/Socio-Economic Indexes for Areas (SEIFA) The Index of Relative Socio-economic Disadvantage (IRSD) (1986) [51]	Australia	1986 Census (Statistical Area Level 3(SA3))	Between 30,000 and 130,000 people	SEIFA1991, SEIFA1996,SEIFA2001,SEIFA2006,SEIFA2011,SEIFA2016[52]	Principal Component Analysis (PCA)	available
**42**	Bell et al. (2007) Vancouver Area Neighborhood Deprivation Index (VANDIX) [53]	Canada (Vancouver)	2001 Census (4000 dissemination areas (DAs))	400-700 people		Expert weighting	available
**43**	Langlois et all. (1996) General Deprivation Index (GDI) [54]	Canada (Montreal)	1996 Census (census tract)	N/A		Principal components analysis (PCA)	
**44**	Pampalon et al. (2000) Deprivation Index for Health and Welfare Planning for Quebec (DIHWPQ) [55]	Canada (Quebec)	1991 Census (enumeration area (EA))	550 people	1996 Quebec index(QI), 2001QI, 2006QI[56]	Principal components analysis (PCA)	available

(Continued)

#	Name of index/reference (year of publication)	Country (area of a country)	Data source (reporting unit)	Population per Unit (average)	Updates (Author/year of publication)	Weighting method	Website and/or supplementary documentation
**45**	Mustard et al. (1995) Socio-Economic Risk Index (SERI)(1995)/Socio-economic Factor Index (SEFI) [57]	Canada (Manitoba)	1986 Census (1,825 enumeration areas)	Maximum of 440 people	SEFI91, SEFI96, SEFI01, Metge et al. (2009) [58] SEFI-2, Bell et al. (2007) [59] Order Weighted Average (OWA) index	Multiple linear regression	available
**46**	Butler et al. (2012) Social Deprivation Index (SDI) [60]	USA	2005–2009 American Community Survey (ACS) 5-year estimates (census tract)	4,000 residents		Principal components analysis (PCA)	available
**47**	Andrews et al. (2020) Neighborhood Deprivation Index (NDI) [61]	USA	2010 Census (census tract)	4,823 -3,532 people		Principal components analysis (PCA)	
**48**	Singh et al. (2002) Factor-Based Area Socioeconomic Index [62]	USA	2010 Census (2,952 counties)	150,000-500,000 people	Kind et al.(2018)[63] Area deprivation index	Principal components analysis (PCA)	available
**49**	Diez-Roux et al. (2001) Neighborhood Socioeconomic Index [64]	USA	2010 Census (census tract)	4,000 residents		Equal weighting	
**50**	Sampson et al. (1997) Deprivation Index [65]	USA	1990 Census (343 "neighborhood clusters" (NCs))	8,000 people		Principal components analysis (PCA)	

(Continued)

#	Name of index/reference (year of publication)	Country (area of a country)	Data source (reporting unit)	Population per Unit (average)	Updates (Author/year of publication)	Weighting method	Website and/or supplementary documentation
**51**	Perkins et al. (2012) Health Opportunity Index (HOI) [66]	USA	Census (census tract)	4,000 residents		Principal components analysis (PCA)	available
**52**	Messer et al. (2006) Deprivation Index [67]	USA	2000 Census (census tract)	4,000 residents		Principal components analysis (PCA)	
**53**	Lòpez-De Fede et al.(2016) Palmetto Small-Area Deprivation Index (Palmetto SADI) [68]	USA (Palmetto)	2000 Census (424 ZIP Code Tabulation Areas (ZCTAs))	N/A		Equal weighting	
**54**	Brokamp et al. (2016) Deprivation Index [69]	USA	2006-2010 American Community Survey (census tract)	4,000 residents	a 2015 Deprivation Index[70]	Principal components analysis (PCA)	
**55**	Wheeler et al. (2019) Neighborhood Deprivation Index [71]	USA	2005–2009 American Community Survey (census tract)	4,000 residents		Bayesian factor analysis	
**56**	Kolak et al. (2020) Multidimensional Social Determinants of Health (SDOH) index [72]	USA	American Community Survey (71,901 census tracts)	2,500 and 8,000 persons		Principal components analysis (PCA)	
**57**	Dubowitz et al. (2008) Neighborhood Socioeconomic Status (NSES) Index [73]	USA	American Community Survey (census tract)	4,000 residents		Exploratory factor analysis	

(Continued)

#	Name of index/reference (year of publication)	Country (area of a country)	Data source (reporting unit)	Population per Unit (average)	Updates (Author/year of publication)	Weighting method	Website and/or supplementary documentation
**58**	Peyvandi et al. (2020) Environmental Exposure Index (EEI) [74]	USA (California)	California Communities Environmental Health Screening Tool, version 3.0 (CalEnviroScreen) (census tract)	N/A		Equal weighting	
**59**	Maizlish et al. (2019) California Healthy Places Index (HPI) [75]	USA (California)	2011-2015 American Community Survey (census tract)	4,000 residents		Weighted Least Squares Regression	available
**60**	Acevedo-Garcia et al. (2014) Child Opportunity Index [76]	USA	Child Opportunity Index 2.0 database (census tract)	N/A		Equal weighting	

Table S2. Indicators of deprivation measures.

#	Name of index/reference (year of publication)	Variables/ subdimensions	Domains/ dimensions	Type of deprivation
**1**	Zadnik et al. (2006) Deprivation Index (DI) [1]	Employment/Occupation<br>Number of unemployed persons per 100 inhabitants<br>Average earning per capita<br>Education<br>Number of graduate students per 100 inhabitants in age group 20–24<br>Number of inhabitants having more than secondary school per 100 inhabitants<br>Number of inhabitants having at least primary school per 100 inhabitants<br>Family structure/ Demographics<br>Natural increase per 1000 inhabitants<br>Number of families living with both parents per 100 families<br>Housing<br>Number of households owing its own bathroom per 100 households<br>Number of households with central heating per 100 households<br>Number of members of an average households	Employment/Occupation<br>Education<br>Family structure/ Demographics<br>Housing	Social, Material
**2**	Meijer M. et al. (2013) Danish Deprivation Index (DANDEX) [2]	Transportation<br>Proportion of households(PH) with no car<br>Housing<br>PH being rented<br>Employment/Occupation<br>Proportion of inhabitants(PI) on unemployment benefits<br>PI with manual profession<br>Family structure/ Demographics<br>PI living alone with child/children<br>Social environment<br>PI with criminal record<br>Education<br>PI with basic education	Transportation<br>Housing<br>Employment/Occupation<br>Family structure/ Demographics<br>Social environment<br>Education<br>Income and Wealth	Social, Material

		Income and Wealth<br>PI with low disposable income		
**3**	Benmarhnia et al. (2014) Spatial Environmental Deprivation (SED) Index [3]	Air quality<br>Mean of PM10 yearly concentrations for all monitoring stations<br>Proportion of the urban population who lives in areas exceeding who standards for SO_2 daily concentrations<br>Proportion of the urban population who lives in areas exceeding who standards for O_3 daily concentrations<br>Proportion of the urban population who lives in areas exceeding who standards for PM10 yearly concentrations<br>Water quality<br>Percentage of drinking water bacterial inspections that found non-conformity with French guidelines<br>Industrial risks<br>Number of industrial facilities presenting medium-level environmental risks per capita<br>Number of industrial facilities presenting high-level environmental risks per capita<br>Number of sites with polluted soil per capita<br>Percentage of industrial facilities mandated to have a safety plan that have actually registered and approved safety plans<br>Proportion of cities with at least one hazardous industrial facility<br>Noise<br>Number of airport noise plans per capita<br>Indoor environment<br>Percentage of housing units built before 1949<br>Number of orders dealing with insalubrious housing per capita<br>Mean radon concentrations measured in public buildings (standardized measurement)	Air quality<br>Water quality<br>Industrial risks<br>Noise<br>Indoor environment	Environmental
**4**	Havard et al. (2008) Havard Index [4]	Employment/Occupation<br>Blue-collar workers in the labour force<br>People in the labour force with insecure jobs<br>People in the labour force with stable jobs<br>Unemployed people in the labour force	Employment/Occupation<br>Housing<br>Transportation<br>Education	Social, Material

		People in the labour force unemployed for more than 1 year<br>Housing<br>Primary residences that are houses or farms<br>Primary residences that are multiple dwelling units<br>Primary residences with more than one person per room<br>Non-owner-occupied primary residences<br>Subsidised housing among all primary residences<br>Mean number of people per room<br>Transportation<br>Households without a car<br>Households with two or more cars<br>Education<br>People aged 15 years or older with general or vocational maturity certificates<br>People aged 15 years or older with at least a lower tertiary education<br>People aged 15 years or older who did not go beyond an elementary education<br>Family structure/ Demographics<br>Single-parent families<br>Income and Wealth<br>Median income per consumption unit (in euros per year)<br>Immigration<br>Foreigners in the total population	Family structure/ Demographics<br>Income and Wealth	
**5**	Rey et al. (2009) French Deprivation Index (FDep) FDep99 Index [5]	Income and Wealth<br>Median household income<br>Education<br>Percentage high school graduates in the population aged 15 years and older<br>Employment/Occupation<br>Percentage blue-collar workers in the active population<br>Unemployment rate	Income and Wealth<br>Education<br>Employment/Occupation	Social, Material
**6**	Lasbeur et al. (2006) Deprivation Index [7]	Employment/Occupation<br>Proportion of workers (%)<br>Proportion of executives (%)	Employment/Occupation<br>Education<br>Transportation	Social, Material

		Share of employments with contrat à durée indéterminée (CDI): contracts with an unlimited term (%)<br>Unemployment rate (%)<br>Education<br>Proportion of people having a primary level of studies (%)<br>Transportation<br>Share of households with no car (%)<br>Family structure/ Demographics<br>Proportion of families single parents (%)<br>Housing<br>Average number of people per room<br>Immigration<br>Proportion of people born abroad (%)	Family structure/ Demographics<br>Housing<br>Immigration	
**7**	Hagedoorn et al. (2016) Municipal Level Deprivation Index [9]	Employment/Occupation<br>Percentage unemployed among men aged 18–64 in the municipality<br>Transportation<br>Percentage of households without a car<br>Education<br>Percentage inhabitants aged 25–64 who are lower educated (lower secondary education or less)	Employment/Occupation<br>Transportation<br>Education	Social, Material
**8**	Splichalova et al. (2007) Index of Socio-Economic Deprivation (SESDI) INDEX1 [10]	Housing<br>Other than detached houses (%)<br>Density of housing (m2/person)<br>No cottage houses (%)<br>Transportation<br>No car (%)<br>Communication<br>No phone (%)<br>Employment/Occupation<br>Unemployment (%)<br>Family structure/ Demographics<br>Single women (%)<br>Single men (%)<br>Education	Housing<br>Transportation<br>Communication<br>Employment/Occupation<br>Family structure/ Demographics<br>Education	Social, Material

		Basic education (%)		
**9**	Winkleby et al. (2007) Neighborhood-Level Deprivation Index [11]	<u>Education</u><br>Low educational status (<10 years of formal education)<br><u>Employment/Occupation</u><br>Unemployment (not employed, excluding full-time students, those completing compulsory military service, and early retirees)<br><u>Income and Wealth</u><br>Low income (income from all sources, including that from interest and dividends, defined as less than 50% of individual median income)<br>Social welfare recipient (receiving social welfare support during 1995)	Education<br>Employment/Occupation<br>Income and Wealth	Social, Material
**10**	Ruiz-Ramos et al. (2006) Socio-Economic Synthetic Index [12]	<u>Education</u><br>Low level of education (people who cannot read or write or with less than 5 years of study / population aged 16 and over)<br><u>Employment/Occupation</u><br>Unemployed (population aged 16 and over unemployed / population aged 16 and over active)<br>Unskilled workers (employed population aged 16 and over unskilled / employed population aged 16 and over)	Education<br>Employment/Occupation	Social, Material
**11**	Salcedo et al. (2012) Deprivation Index (DI) [13]	<u>Employment/Occupation</u><br>Unemployment rate<br>Manual workers<br>Temporary workers<br>Activity rate<br><u>Education</u><br>Percent with low educational level<br>University education<br><u>Family structure/Demographics</u><br>Mono-parental homes<br><u>Immigration</u><br>Immigrants<br><u>Housing</u><br>Homes without heating<br>No toilet/bathroom in the home	Employment/Occupation<br>Education<br>Family structure/ Demographics<br>Immigration<br>Housing<br>Communication<br>Social environment<br>Green space<br>Noise<br>Air quality	Social, Material, Environmental

		<u>Communication</u><br>Bad communications<br><u>Social environment</u><br>Vandalism/crime<br>Dirty street<br><u>Green space</u><br>Few green areas<br><u>Noise</u><br>Exterior noise<br><u>Air quality</u><br>Contamination/bad smells		
**12**	Dominguez-Berjon et al. (2008) MEDEA Index [14]	<u>Employment/Occupation</u><br>Manual workers 16 years : percentage of people aged 16 or over employed, who are manual workers<br>Unemployment 16 years: percentage of unemployed persons aged 16 or over<br>Casual wage earners 16 years: percentage of employed persons aged 16 or more years they are temporary employees<br><u>Education</u><br>Total insufficient education: percentage of people aged 16 and over who are illiterate or have incomplete primary education<br>Insufficient education in young people: percentage of people aged 16-29 who are illiterate or have incomplete primary education	Employment/Occupation<br>Education	Social, Material
**13**	Compés Dea et al. (2018) Deprivation Index [15]	<u>Employment/Occupation</u><br>%unemployment<br>%eventual workers<br><u>Education</u><br>% insufficient education 16-64 years old<br>% insufficient education in foreign population	Employment/Occupation<br>Education	Social, Material
**14**	Duque et al. (2019) Spanish Deprivation Index (SDI) [16]	<u>Employment/Occupation</u><br>Manual and temporary workers<br>Unemployment<br><u>Education</u><br>Population with insufficient education	Employment/Occupation<br>Education<br>Communication	Social, Material

		Young population (aged 16 to 29 years) with insufficient education<br>Communication<br>Dwellings without access to the interne		
**15**	Colls et al. (2020) Composed Socioeconomic Index (CSI) [17]	Health<br>Drug benefits<br>Population exempt from pharmaceutical copayment<br>Mortality<br>Mortality prior to 75 years<br>Hospitalization<br>Potentially avoidable hospitalizations(number of potentially avoidable hospitalizations for COPD, heart failure, and diabetes complications/Population older than 19 years)<br>Income and Wealth<br>Income below € 18,000<br>Income above € 100,000<br>Employment/Occupation<br>Manual occupations<br>Education<br>Insufficient education level	Health:<br>• Drug benefits<br>• Mortality<br>• Hospitalization<br>Income and Wealth<br>Employment/Occupation<br>Education	Social, Material, Health
**16**	Álvarez-del Arco et al. (2013) Deprivation Index (DI) [18]	Employment/Occupation<br>Unemployment rate or activity rate<br>Education<br>Percentage of the illiterate population + without studies or with third grade studies<br>Housing<br>Average number of rooms<br>Average surface of a house<br>Percentage of homes without heating<br>Percentage of population in dwellings without heating<br>Percentage of population in rental housing<br>Second hand home price<br>Transportation<br>Percentage of population with only one car<br>Percentage of the population that has more than one car	Employment/Occupation<br>Education<br>Housing<br>Transportation<br>Immigration	Social, Material

		Number of cars<br>Immigration<br>Immigration rate		
**17**	Ocaña-Riola et al. (2008) Material Deprivation Index [19]	Employment/Occupation<br>Manual labourers (the number of workers occupied as skilled construction workers, skilled mine and metal workers, skilled workers in other industries, operators of facilities and machinery, unskilled workers and other employees in the services industry per 100 people in employment)<br>Unemployment (the number of unemployed per 100 persons in the labour force)<br>Education<br>Illiteracy (the number of illiterate individuals per 100 people over the age of 10 years)	Employment/Occupation<br>Education	Social, Material
**18**	Benach et al. (1999) Deprivation Index [20]	Education<br>Illiteracy (percentage of illiterate people in the population over age 10)<br>Employment/Occupation<br>Social class (the number of persons with unskilled occupations divided by all people who ever worked)<br>Unemployment (percentage of unemployed in the population aged 15 to 64 years)<br>Housing<br>Overcrowding (percentage of households with more than one person per room)	Education<br>Employment/Occupation<br>Housing	Social, Material
**19**	Sánchez-Cantalejo et al. (2008) Deprivation Index [21]	Employment/Occupation<br>Number of unemployed per 100 active people<br>Number of artisans and skilled workers in the manufacturing, construction and mining industries, operators of installations and machinery, installers and fitters and non-skilled workers per 100 workers<br>Education<br>Number of illiterate persons above the age of 10 per 100 people above the age of 10<br>Transportation<br>Number of homes without access to a vehicle per 100 homes<br>Family structure/Demographics	Employment/Occupation<br>Education<br>Transportation<br>Family structure/ Demographics<br>Immigration	Social, Material

		Number of persons older than 64 divided by the number of persons younger than 15<br><u>Immigration</u><br>Number of foreigners between 16 and 49 who lived abroad in 1991 per 100 foreigners		
**20**	García-Gil et al. (2004) Social Index [22]	<u>Employment/Occupation</u><br>Unemployment among males aged 16 + years<br><u>Education</u><br>The rate of illiteracy, standardized by age, in females (10–64 years of age)<br>The percentage of females (25–64 years of age) in work and of at least secondary-level education (standardized by age)<br>The percentage of males and females (aged 25 + years) with university-level education	Employment/Occupation<br>Education	Social, Material
**21**	Esnaola et al. (2006) Deprivation Index [23]	<u>Employment/Occupation</u><br>Proportion in unemployment<br>Proportion of unskilled manual workers<br><u>Education</u><br>Proportion with primary or lower educational level<br><u>Housing</u><br>Proportion of low-comfort housing	Employment/Occupation<br>Education<br>Housing	Social, Material
**22**	Panczak et al. (2012) Swiss Neighborhood Index of Socio-Economic Position (Swiss-SEP) [24]	<u>Education</u><br>Proportion households headed by a person with primary education or less<br><u>Employment/Occupation</u><br>Proportion headed by a person in manual or unskilled occupation<br><u>Housing</u><br>Median rent per square meter<br>The mean number of persons per room	Education<br>Employment/Occupation<br>Housing	Social, Material
**23**	Juhász et al. (2010) Hungarian Deprivation Index [25]	<u>Income and Wealth</u><br>Gross income serving as the basis for the personal income tax per person as a member of the permanent population<br><u>Education</u><br>Proportion of the population with basic education and illiteracy in the total population older than 15 years<br><u>Employment/Occupation</u>	Income and Wealth<br>Education<br>Employment/Occupation<br>Family structure/ Demographics<br>Housing<br>Transportation	Social, Material

		Unemployment (as a proportion of total population of productive age)<br>Family structure/Demographics<br>One-parent families (single mothers with children, as a proportion of families)<br>Large families (parents with three or more children, as a proportion of families)<br>Housing<br>Density of housing (persons per room)<br>Transportation<br>Car (passenger cars per 100 inhabitants)		
**24**	Caranci et al. (2010) Italian Deprivation Index [26]	Education<br>Low level of education<br>Employment/Occupation<br>Unemployment<br>Housing<br>Non-home ownership<br>Overcrowding<br>Family structure/Demographics<br>One parent family	Education<br>Employment/Occupation<br>Housing<br>Family structure/ Demographics	Social, Material
**25**	Ivaldi et al. (2001) Genoa Deprivation Index [28]	Housing<br>House ownership (% of households living in rented houses)<br>Household overcrowding(average number of occupants per room)<br>Employment/Occupation<br>Unemployment rate (% of unemployed people compared to total workforce)<br>Education<br>Low education level (% of people with a degree lower or equal to middle school diploma)	Housing<br>Employment/Occupation<br>Education	Social, Material
**26**	Cadum et al. (1999) National deprivation index [29]	Education<br>Proportion of the population educated with an elementary school certificate<br>Housing<br>Proportion of rented dwellings<br>Proportion of occupied dwellings without bathroom inside the house	Education<br>Housing<br>Employment/Occupation<br>Family structure/ Demographics	Social, Material

		<u>Employment/Occupation</u><br>Proportion of the active population unemployed or seeking their first job<br><u>Family structure/Demographics</u><br>Proportion of single-parent families with children		
**27**	Michelozzi et al. (1998) Deprivation Index [30]	<u>Education</u><br>Percentage of people by educational level<br><u>Employment/Occupation</u><br>Percentage of people employed by occupational category<br>Percentage of unemployed men of working age<br><u>Family structure/Demographics</u><br>Percentage of one person families<br>Percentage of families with five or more persons<br><u>Housing</u><br>Crowding index (persons/room),<br>Percentage of dwellings rented or owned	Education<br>Employment/Occupation<br>Family structure/ Demographics<br>Housing	Social, Material
**28**	Petrelli et al. (2006) Deprivation Index [31]	<u>Education</u><br>Low educational level (primary school or less)<br><u>Employment/Occupation</u><br>Manual job<br><u>Housing</u><br>Rented dwellings<br>Dwellings without an indoor bathroom<br>Household crowding<br><u>Family structure/Demographics</u><br>Single-parent households with children	Education<br>Employment/Occupation<br>Housing<br>Family structure/ Demographics	Social, Material
**29**	Jordan et al. (2004) Index of Multiple Deprivation 2000 [32]	<u>Income and Wealth</u><br>Adults in income support households<br>Children in income support households<br>Adults in income based job seekers allowance households<br>Children in income based job seekers<br>Allowance households<br>Adults in family credit households<br>Children in family credit households<br>Adults in disability working allowance households	Income and Wealth<br>Employment/Occupation<br>Health:<br>• Mortality<br>• Disability<br>• Birth rate<br>Education<br>Housing<br>Build environment	Social, Material, Health, Environment al

Children in disability working allowance households
Non-earning, non is pensioner and disabled council tax
Benefit recipients
<u>Employment/Occupation</u>
Unemployment claimant counts
People out of work but in TEC delivered
Government supported training
People aged 18–24 on New Deal options
Incapacity benefit recipients of working age
Severe Disablement Allowance claimants of working age
<u>Health</u>
<u>Mortality</u>
Comparative mortality ratio for men and women aged under 65
<u>Disability</u>
People receiving attendance allowance or disability living allowance
People of working age receiving incapacity
Benefit or Severe Disablement Allowance
Age-sex standardized ratio of Limiting Long Term Illness (LLTI)
<u>Birth rate</u>
Proportion of births of low birth weight
<u>Education</u>
Working age adults with no qualifications
Children aged 16+ not in full time education
Proportion of 17–19 year olds who have not successfully
Applied to higher education
KS2 primary school performance data
Primary school children with English as an additional language
Absenteeism at primary level
<u>Housing</u>
Homeless households in temporary accommodation
Household overcrowding
Poor private sector housing
<u>Build environment</u>

		Access to a post office<br>Access to food shops<br>Access to a GP<br>Access to a primary school		
**30**	Townsend et al. (1988) Townsend Material Deprivation Score [34]	Employment/Occupation<br>Percentage of economically active residents aged 16-59 or 16-64 who are unemployed<br>Housing<br>Percentage of private households that are not owner occupied<br>Percentage of private households with >1 person per room<br>Transportation<br>Percentage of private households that do not possess a car	Employment/Occupation<br>Housing<br>Transportation	Material
**31**	Jarman (1983) Jarman Score [35]	Family structure/Demographics<br>Families of non-married couples (less stable family groups)<br>Children aged <5<br>Single parent households<br>Elderly people living alone<br>Employment/Occupation<br>Unemployment<br>Lower social classes<br>Immigration<br>Ethnic minorities (people born outside United Kingdom)<br>Housing<br>Overcrowded households<br>Poor housing<br>Mobility<br>Highly mobile people (percentage changing house in a year)	Family structure/ Demographics<br>Employment/Occupation<br>Housing<br>Mobility<br>Immigration	Social, Material
**32**	Carstairs et al. (1991) Carstairs Deprivation Index [36]	Employment/Occupation<br>Male unemployment (economically active males aged 16–74 seeking or waiting to start work, as a proportion of all economically active males 16–74)<br>Low social class (persons living in households with the	Employment/Occupation<br>Housing<br>Transportation	Material

		household reference person (HRP) (aged 16–74) in social class IV or V, as a proportion of all people in households with an economically active HRP (16–74))<br><u>Housing</u><br>Overcrowding (persons living in households at a density of more than one person per room, as a proportion of all people in households)<br><u>Transportation</u><br>No car ownership (persons living in households with no car, as a proportion of all people in private households)		
**33**	Simpson et al. (1994) Index of Local Conditions (ILC) [37]	<u>Employment/Occupation</u><br>Unemployment<br>Long-term unemployment<br><u>Education</u><br>Low educational participation aged 17<br>Low educational attainment<br><u>Housing</u><br>Home insurance weightings<br>Households lacking amenities<br>Overcrowded housing<br><u>Transportation</u><br>Households lacking a car (income proxy)<br><u>Family structure/Demographics</u><br>Children in unsuitable accommodation<br>Children in low earning households<br><u>Income and Wealth</u><br>Income support<br><u>Health</u><br><u>Mortality</u><br>Standardized mortality ratios<br><u>Green space</u><br>Derelict land	Employment/Occupation<br>Education<br>Housing<br>Transportation<br>Family structure/Demographics<br>Health:<br>• Mortality<br>Income and Wealth<br>Green space	Social, Material, Health, Environmental
**34**	Richardson et al. (2010) Multiple Environmental	<u>Air quality</u><br>Particulate matter (PM10) 10 micrometers or less in diameter<br>Ozone (O_3)	Air quality<br>Climate<br>UV radiation	Environmental

	Deprivation Index (MEDIx) [39]	Nitrogen dioxide (NO_2)<br>Sulphur dioxide (SO_2)<br>Carbon monoxide (CO)<br><u>Climate</u><br>Average temperature<br>Cooling degree-days1<br>Heating degree-days2<br>Winter cold wave duration<br>Summer heatwave duration<br><u>UV radiation</u><br>UV radiation<br><u>Industrial risks</u><br>Waste management sites<br>Metal production/processing sites<br><u>Green space</u><br>Green space	Industrial risks<br>Green space	
**35**	Noble et al. (2001) Northern Ireland Multiple Deprivation Measure (NIMDM) [40]	<u>Income and Wealth</u><br>Adults in income support households<br>Children in income support households<br>Adults in income based job seekers allowance households<br>Children in income based job seekers allowance households<br>Adults in family credit households<br>Children in family credit households<br>Adults in disability working allowance households<br>Children in disability working allowance households<br><u>Employment/Occupation</u><br>Unemployment claimant counts of those aged under 60 (average of four quarterly extracts)<br>Incapacity benefit recipients aged under 60<br>Severe disablement allowance recipients aged under 60<br>New deal participants not included in the unemployment claimant count<br>Working age adults with no qualifications<br><u>Health</u><br><u>Mortality</u>	Income and Wealth<br>Employment/Occupation<br>Health:<br>• Mortality<br>• Disability<br>• Cancer<br>• Dentistry<br>• Mental disorders<br>Education<br>Housing<br>Build environment<br>Social Environment	Social, Material, Health, Environmental

Standardized mortality ratios for men and women at ages under 75
Disability
People receiving one or more of attendance allowance or disability living Allowance or incapacity benefit or severe disablement allowance
Cancer
People registered as having cancer (excluding non-melanoma skin cancers)
Dentistry
Proportion of all 12 to 17 year olds with extractions and registered with a GDS dentist, and those not registered with a GDS dentist
Mental disorders
Drugs prescribed for depression or anxiety
Education
Proportions of those leaving school aged 16 and not entering further education
Proportions of 17-20 year olds who have not successfully applied for higher education
GCSE/GNVQ performance data points score and no qualifications
Absenteeism at secondary level
Proportions of years 11 and 12 pupils not in a grammar school
Housing
Housing in disrepair
Houses without central heating
Houses lacking insulation
Build environment
Access to a post office
Access to a GP surgery
Access to an accident and emergency hospital
Access to a dentist
Access to an optician
Access to a pharmacist
Access to a library
Access to a museum
Access to a social security office or a training and employment agency
Social Environment

		Burglary in a dwelling<br>Violence against the person (excluding assaults)<br>Common assaults<br>Serious assaults<br>Theft of a vehicle<br>Theft from a vehicle<br>Criminal damage<br>Burglary in a building other than a dwelling<br>Drug offences local area problem score (graffiti, scruffy/neglected buildings, scruffy gardens/landscaping, litter/rubbish/dumping and vandalism)		
**36**	Kelly et al.(1997) Small Area Health Research Unit (SAHRU) Deprivation Index [42]	<u>Employment/Occupation</u><br>Unemployment<br>Low social class<br><u>Transportation</u><br>No car<br><u>Housing</u><br>Rented accommodation<br>Overcrowding	Employment/Occupation<br>Transportation<br>Housing	Material
**37**	Haase, Pratschke and Gleeson (2014) All-Island Deprivation Index [44]	<u>Family structure/Demographics</u><br>Percentage change in population over the previous five years<br>Percentage of people aged under 15 or over 64 years of age<br>Percentage of households with children aged under 15 years and headed by a single parent<br><u>Education</u><br>Percentage of people with a primary school education only<br>Percentage of people with a third-level education<br>Percentage of people with a primary school education only<br>Percentage of people with a third-level education<br><u>Housing</u><br>Mean number of persons per room<br><u>Employment/Occupation</u><br>Percentage of households headed by professionals or managerial and technical employees, including farmers with 100 acres or more	Family structure/ Demographics<br>Education<br>Housing<br>Employment/Occupation	Social, Material

		Percentage of households headed by semiskilled or unskilled manual workers, including farmers with less than 30 acres<br>Male unemployment rate<br>Female unemployment rate		
**38**	Welsh Index of Multiple Deprivation (WIMD) (2000) [45]	<u>Income and Wealth</u><br>Percentage of population in income deprivation those who are: in receipt of income related benefits; in receipt of tax credits with and income 60% below the wales median; or a supported asylum seeker<br><u>Employment/Occupation</u><br>Percentage of working age population in receipt of employment related benefits<br><u>Health</u><br><u>Mortality</u><br>All-cause death rate<br><u>Cancer</u><br>Cancer incidence long term<br><u>Disability</u><br>Limiting illness<br><u>Birth rate</u><br>Low birth weight<br><u>Education</u><br>Key stage 2 average point score<br>Key stage 4 capped point score<br>Key stage 4 level 2 inclusive<br>Repeat absenteeism<br>Proportion of 18-19 year olds not entering higher education<br>Proportion of 25-64 year olds with no qualifications<br><u>Build environment</u><br>Average travel time by public and private transport to the nearest: food shop, general practitioner (GP) surgery, post office, public library, leisure centre, primary school, secondary school, pharmacy, and petrol station (private transport only)<br><u>Social environment</u>	Income and Wealth<br>Employment/Occupation<br>Health:<br>• Mortality<br>• Cancer<br>• Disability<br>• Birth rate<br>Housing<br>Education<br>Social environment<br>Build environment<br>Air quality<br>Natural disaster<br>Industrial risks	Social, Material, Health, Environment al

		Police recorded violent crime<br>Police recorded criminal damage<br>Police recorded burglary<br>Police recorded theft<br>Antisocial behaviour<br>Fire incidence<br>Air quality<br>Air concentrations<br>Air emissions<br>Natural disaster<br>Flood risk<br>Industrial risks<br>Proximity to waste and industrial sites<br>Housing<br>Overcrowding (bedroom occupancy)<br>Lack of central heating		
**39**	Scottish Index of Multiple Deprivation (SIMD) (2004) [47]	Income and Wealth<br>Adults in income support households<br>Children (aged 0-19) in income support households<br>Adults in income based job seekers allowance households<br>Children in (aged 0-19) income based job seekers allowance households<br>Adults in working families tax credit<br>Households below a low income threshold<br>Children in working families tax credit<br>Households below a low income threshold<br>Adults in disability tax credit households below a low income threshold<br>Children in disability tax credit households below a low income threshold<br>Employment/Occupation<br>Unemployment claimant count averaged over 12 months of those men aged under 65 and women aged under 60<br>Incapacity benefit recipients, men aged under 65 and women aged under 60<br>Severe disablement allowance recipients, men aged under 65 and women aged under 60	Income and Wealth<br>Employment/Occupation<br>Health:<br>• Mortality<br>• Disability<br>• Bad habit<br>• Hospitalization<br>• Mental disorders<br>• Birth rate<br>Education<br>Build environment<br>Housing	Social, Material, Health, Environmental

		Compulsory new deal participants — new deal for the under 25s and new deal for the 25+ not included in the unemployment claimant count<br><u>Health</u><br><u>Mortality</u><br>Comparative mortality factor<br><u>Bad habits</u><br>Hospital episodes related to alcohol use<br>Hospital episodes related to drug use<br><u>Disability</u><br>Comparative illness factor<br><u>Hospitalization</u><br>Emergency admissions to hospital<br><u>Mental disorders</u><br>Proportion of population being prescribed drugs for anxiety, depression or psychosis<br><u>Birth rate</u><br>Proportion of live singleton births of low birth weight<br><u>Education</u><br>Secondary level absences<br>Pupil performance on SQA at Stage 4<br>Working age adults with no qualifications<br>Proportion of the 17+ population not applying successfully to HE<br>Pupils age 16+ not in full time education<br><u>Build environment</u><br>Drive time to a GP<br>Drive time to a petrol station<br>Drive time to a post office<br>Drive time to a primary school<br>Drive time to a supermarket<br><u>Housing</u><br>Persons in households that are overcrowded<br>Persons in households without central heating		
**40**	Salmond et al. (2012) New	<u>Income and Wealth</u>(*(NZDep91))<br>Equivalised* household income below an income threshold	Income and Wealth<br>Housing	Social, Material

	Zealand's Deprivation Index (NZDep) [49]	Equivalised* household below an occupancy threshold<br>Means tested benefit and age 18-59<br><u>Housing</u><br>Not living in own home<br><u>Transportation</u><br>Adults with no access to a car<br><u>Employment/Occupation</u><br>Unemployed and age 18-59<br>No qualifications and age 18-59<br><u>Family structure/Demographics</u><br>Single parent family<br>Separated or divorced and age 18-59<br>Separated or divorced and age 60 plus	Transportation<br>Employment/Occupation<br>Family structure/ Demographics	
**41**	Ross index/Socio-Economic Indexes for Areas (SEIFA) The Index of Relative Socio-economic Disadvantage (IRSD) (1986) [51]	<u>Income and Wealth</u><br>% of people with stated household equivalised income between $1 and $25,999 per year<br><u>Employment/Occupation</u><br>% of families with children under 15 years of age who live with jobless parents<br>% of people (in the labour force) who are unemployed<br>% of employed people classified as Labourers<br>% of employed people classified as Machinery Operators and Drivers<br>% of employed people classified as low skill Community and Personal Service workers<br><u>Communication</u><br>% of occupied private dwellings with no internet connection<br><u>Education</u><br>% of people aged 15 years and over whose highest level of education is year 11 or lower<br>% of people aged 15 years and over who have no educational attainment<br><u>Housing</u><br>% of occupied private dwellings paying rent less than $215 per week (excluding $0 per week)<br><u>Family structure/Demographics</u>	Income and Wealth<br>Employment/Occupation<br>Communication<br>Education<br>Housing<br>Family structure/ Demographics<br>Transportation<br>Immigration	Social, Material

		% of one parent families with dependent offspring only<br>% of people under the age of 70 who have a long-term health condition or disability and need assistance with core activities<br>% of people aged 15 years and over who are separated or divorced<br>Transportation<br>% of occupied private dwellings with no cars<br>Housing<br>% of occupied private dwellings requiring one or more extra bedrooms<br>Immigration<br>% of people who do not speak English well		
**42**	Bell et al. (2007)<br>Vancouver Area Neighbourhood Deprivation Index (VANDIX) [53]	Income and Wealth<br>Average income<br>Housing<br>Average dwelling value<br>Single-detached housing<br>Home ownership<br>Proportion of renters<br>Reside in an apartment<br>Employment/Occupation<br>Employment ratio<br>Unemployment rate<br>Females in labour force<br>Family structure/Demographics<br>Elderly 65+living alone<br>Living alone<br>Single parent family<br>Separated/divorced/widowed<br>Children under age 5<br>Family size + 5 persons<br>Mobility<br>Moved in the last 5 years<br>Moved in the last year<br>Education<br>No high school completion	Income and Wealth<br>Housing<br>Employment/Occupation<br>Family structure/ Demographics<br>Mobility<br>Education<br>Immigration	Social,<br>Material

		With a university degree<br>Immigration<br>Non-Canadian citizen<br>First language non-official		
**43**	Langlois et al. (1996) General Deprivation Index (GDI) [54]	Family structure/Demographics<br>Rate of population change from 1991 to 1996<br>Percentage of males in the total population<br>Percentage of the total population aged 15– 25 years<br>Percentage of the total population aged 65 years and over<br>Percentage of the total population that is single<br>Percentage of total census families that are headed by a single female parent<br>Income and Wealth<br>Median family income in Canadian dollars<br>Percentage of all economic families living below statistics Canada's low-income cut-off (LICO)<br>Education<br>Percentage of the total population 15 years and over whose highest level of schooling is grade 12 or lower<br>Immigration<br>Percentage of the total population whose mother tongue is a non-official language (neither English nor French)<br>Housing<br>Average value of dwellings in Canadian dollars<br>Percentage of household occupants who are renters<br>Percentage of occupied private dwellings that are in need of major repair<br>Employment/Occupation<br>Percentage of young people aged 15– 25 years who are unemployed<br>Percentage of young people aged 15– 25 years who are in the labour force<br>Percentage of the total male population aged 15 years and over who are unemployed<br>Percentage of the total male population aged 15 years and over who are in the labour force	Family structure/ Demographics<br>Income and Wealth<br>Education<br>Immigration<br>Housing<br>Employment/Occupation<br>Indoor environment	Social, Material, Environmental

		Percentage of the total female population aged 15 years and over who are unemployed<br>Percentage of the total female population aged 15 years and over who are in the labour force<br><u>Indoor environment</u><br>Percentage of occupied private dwellings built before 1960		
**44**	Pampalon et al. (2000)the Deprivation Index for Health and Welfare Planning for Quebec (DIHWPQ) [55]	<u>Education</u><br>The proportion of people aged 15 years and older with no high school diploma<br><u>Employment/Occupation</u><br>The employment/population ratio* of people aged 15 years and older<br><u>Income and Wealth</u><br>The average income of people aged 15 years and older<br><u>Family structure/Demographics</u><br>The proportion of individuals aged 15 years and older living alone<br>The proportion of individuals aged 15 years and older who are separated, divorced or widowed<br>The proportion of single-parent families<br>(* proportion of people among those aged 15 years and older, adjusted according to the age and sex of the Quebec population)	Education<br>Employment/Occupation<br>Income and Wealth<br>Family structure/ Demographics	Social, Material
**45**	Mustard et al. (1995) Socio-Economic Risk Index (SERI)/Socio-economic Factor Index (SEFI) [57]	<u>Employment/Occupation</u><br>Labour force unemployment among persons aged 15-24<br>Unemployment among those aged 45-54<br>Female labour force participation<br><u>Family structure/Demographics</u><br>Percentage of single parent female households<br><u>Education</u><br>Percentage of the population aged 25-34 having graduated high school<br><u>Housing</u><br>The average value of owner-occupied dwellings	Employment/Occupation<br>Education<br>Family structure/ Demographics<br>Housing	Social, Material
**46**	Butler et al. (2012) Social Deprivation Index (SDI) [60]	<u>Income and Wealth</u><br>% living in poverty,<br><u>Racial composition</u><br>% black,	Income and Wealth<br>Racial composition<br>Education<br>Family structure/	Social, Material

		<u>Education</u><br>less than 12 years of schooling,<br><u>Family structure/demographics</u><br>Single parent households<br>The percent of the population under the age of 5<br>Female between the ages of 15 and 44<br>Persons older than 65<br><u>Housing</u><br>Single occupant households<br>% living in overcrowded conditions (more persons in a dwelling unit than number of rooms),<br><u>Transportation</u><br>Percent of households without a car,<br><u>Employment/Occupation</u><br>Percent of 18- to 64-year olds that are unemployed	Demographics<br>Housing<br>Transportation<br>Employment/Occupation	
**47**	Andrews et al. (2020) Neighborhood deprivation Index (NDI) [61]	<u>Employment/Occupation</u><br>% employed in management (% of employed people (age 16 and older) working in management, business, science, and arts)<br>% unemployment (% of people age 16 and older that are unemployed)<br><u>Education</u><br>% high school graduates(population 25 years and older: % with a high school diploma or higher)<br>% bachelor's degree or higher(population 25 years and older: % with a bachelor's degree or higher)<br><u>Communication</u><br>% households without telephone<br><u>Housing</u><br>% households without plumbing<br><u>Income and Wealth</u><br>Household income (median household income in the last 12 months)<br>Home value (median home value)<br>% family poverty<br>% public assistance (% of families receiving public assistance income in the past 12 months)	Employment/Occupation<br>Education<br>Communication<br>Housing<br>Income and Wealth<br>Family structure/<br>Demographics	Social,<br>Material

		% housing units receiving interest/dividends/rental income (% of housing units that are receiving interest, dividends, or net rental income in the past 12 months for households)<br>Family structure/Demographics<br>% female-headed household (% of female-headed households with children under 18)		
**48**	Singh et al. (2002) Factor-Based Area Socioeconomic Index [62]	Education<br>Percentage of population with <9 years of education<br>Percentage of population with at least 12 years of education<br>Employment/Occupation<br>Occupational composition (% employed persons aged ≥16 years in white collar occupations)<br>Unemployment rate (% civilian labor force aged ≥16 years unemployed)<br>Income and Wealth<br>Median family income<br>Income disparity<br>Family poverty rate<br>Family structure/Demographics<br>Single parent household rate<br>Housing<br>Home ownership rate<br>Median home value<br>Median gross rent<br>Household crowding (percentage of households with more than one person per room)<br>Percentage of households without access to plumbing<br>Communication<br>Percentage of households without access to phone<br>Immigration<br>English language proficiency	Education<br>Employment/Occupation<br>Income and Wealth<br>Family structure/ Demographics<br>Housing<br>Communication<br>Immigration	Social, Material
**49**	Diez-Roux et al. (2001) Neighborhood	Income and Wealth<br>Median household income (U.S.$)<br>Percentage persons below poverty<br>Employment/Occupation	Income and Wealth<br>Employment/Occupation<br>Housing	Material

	Socioeconomic Index [64]	Percentage employed persons 16 years of age and older in executive, managerial or professional occupations<br>Percentage unemployment<br>Housing<br>Percentage households receiving interest, dividend, or net rental income<br>Median value of owner-occupied housing units (U.S.$)<br>Percentage of housing units that are owner occupied<br>Percentage of housing units unoccupied<br>Percentage of housing units boarded up*(* Boarded-up status was obtained for all vacant housing units. Boarded-up units have windows and doors covered by wood, metal, or masonry to protect the interior and to prevent entry into the building.)<br>Percentage occupied housing units with one person per room		
**50**	Sampson et al. (1997) Deprivation Index [65]	Income and Wealth<br>Below poverty line<br>On public assistance<br>Employment/Occupation<br>Unemployed<br>Family structure/Demographics<br>Female-headed families<br>Less that age 18<br>Housing<br>Owner-occupied house<br>Racial composition<br>% black<br>% latino<br>Immigration<br>% foreign-born<br>Mobility<br>Same houses as in 1985	Income and Wealth<br>Employment/Occupation<br>Family structure/ Demographics<br>Housing<br>Racial composition<br>Immigration<br>Mobility	Social, Material
**51**	Perkins et al. (2012) Health	Income and Wealth	Income and Wealth<br>Employment/Occupation	Social, Material,

Opportunity Index (HOI) [66]	Affordability (the proportion of a neighborhood's income that is spent on housing and transportation)<br>Income inequality (the Gini coefficient(index))<br><u>Employment/Occupation</u><br>Unemployment(the percentage of active residents in a community between the ages of 16 to 64 who are unemployed)<br>Job participation (the percentage of working aged individuals (16–64 years of age) in the active labor force),<br>Employment access (the accessibility of jobs in a particular area)<br><u>Transportation</u><br>Car ownership (the percentage of private households who do not possess a car)<br><u>Housing</u><br>Home ownership (the percentage of private homes not currently occupied by the homeowner)<br>Overcrowding<br><u>Education</u><br>Education (the average education level achieved by the adult population in an area)<br><u>Family structure/Demographics</u><br>Population churning<br>Population-weighted density<br><u>Racial composition</u><br>Segregation index measures how the racial composition of a population in a census tract compares to that of the rest of the state<br><u>Build environment</u><br>Food accessibility<br>Walkability<br>Access to care<br><u>Air quality</u><br>Environmental quality index (neurological risk, cancer risk, respiration risk, on-road pollution, non-road pollution, and non-point pollution)	Transportation<br>Housing<br>Education<br>Family structure/ Demographics<br>Racial composition<br>Build environment<br>Air quality	Environmental

**52**	Messer et al. (2006) Deprivation Index [67]	<u>Housing</u><br>Percent of crowded housing,<br><u>Income and Wealth</u><br>Percent of households in poverty,<br>Percent of households on public assistance and households earning <$30,000 per year estimating poverty<br><u>Education</u><br>Percent earning less than a high school education<br><u>Family structure/Demographics</u><br>Percent of female headed households with dependents<br><u>Employment/Occupation</u><br>Percent unemployed<br>Percent of males in management and professional occupations	Housing<br>Income and Wealth<br>Education<br>Family structure/ Demographics<br>Employment/Occupation	Social, Material
**53**	Lòpez-De Fede et al.(2016) Palmetto Small-Area Deprivation Index (Palmetto SADI) [68]	<u>Education</u><br>% persons 25 and older without a high school (HS) diploma<br>% persons 16–19 not enrolled and not a HS graduate<br><u>Income and Wealth</u><br>% persons below poverty level<br>% households with income < $15,000<br>% renter-occupied households<br><u>Employment/Occupation</u><br>% persons 16 and older unemployed<br>% persons 16–64 working part-time<br><u>Family structure/Demographics</u><br>% persons 15 and older unmarried or separated<br>% single female-headed family households<br><u>Transportation</u><br>% households with no vehicle	Education<br>Income and Wealth<br>Employment/Occupation<br>Family structure /Demographics<br>Transportation	Social, Material
**54**	Brokamp et al. (2016) Deprivation Index [69]	<u>Education</u><br>Fraction that graduated high school<br><u>Income and Wealth</u><br>Fraction of households in poverty<br>Median household income<br>Fraction of population receiving public assisted income	Education<br>Income and Wealth<br>Racial composition	Social, Material

		Fraction of houses that are vacant<br>Median home value<br>Racial composition<br>White fraction of population<br>Black fraction of population		
**55**	Wheeler et al. (2019) Neighborhood Deprivation Index [71]	Income and Wealth<br>Household Gini index of income inequality<br>Percent of households with income below federal poverty level in the past 12 months<br>Percent of households receiving public assistance income in the past 12 months<br>Percent of households receiving cash public assistance or food stamps/SNAP<br>Median household income (U.S. Dollars) in the past 12 months<br>Social security income in the past 12 months<br>Racial composition<br>percent of the population that is African-American<br>Family structure/Demographics<br>Percent female headed households with children present<br>Education<br>Percent of population aged ≥ 25 years with less than high school education<br>Employment/Occupation<br>Percent of unemployed population aged 16 years and over<br>Housing<br>Percent of vacant housing units<br>Percent of renter occupied housing units<br>Percent of households receiving<br>Indoor environment<br>Percent of housing units built in 1939 or earlier<br>Percent of housing units built from 1940–1949	Income and Wealth<br>Racial composition<br>Family structure/ Demographics<br>Education<br>Employment/Occupation<br>Housing	Social, Material
**56**	Kolak et al. (2020) Multidimensional Social Determinants of	Racial composition<br>Minority status<br>Family structure/Demographics<br>Over age 65	Racial composition<br>Family structure /Demographics<br>Health:	Social, Material, Health

	Health (SDOH) Index [72]	Under age 17<br>Single parent<br>Health<br>Disability<br>Disabled<br>Health insurance<br>Uninsured<br>Education<br>No high school<br>Immigration<br>Limited English<br>Income and Wealth<br>Poverty<br>Per capita income<br>Employment/Occupation<br>Unemployment<br>Housing<br>The proportion of renters<br>Rent burden (more than 30% of income used for rent payments)<br>Crowded housing conditions (occupied housing units consisting of more people than rooms)<br>Transportation<br>No vehicle	• Disability<br>• Health insurance<br>Education<br>Immigration<br>Income and Wealth<br>Employment/Occupation<br>Housing<br>Transportation	
**57**	Dubowitz et al. (2008) Neighborhood Socioeconomic Status (NSES) Index [73]	Education<br>Percent of adults older than 25 with less than a high school education<br>Employment/Occupation<br>Percent male unemployment<br>Income and Wealth<br>Percent of households with income below the poverty line<br>Percent of households receiving public assistance<br>Median household income<br>Family structure/Demographics<br>Percent of households with children that are headed only by a female	Education<br>Employment/Occupation<br>Income and Wealth<br>Family structure/ Demographics	Social, Material

**58**	Peyvandi et al. (2020) Environmental Exposure Index (EEI) [74]	Industrial risks<br>Toxic release from facilities<br>Air quality<br>Air quality measured by ozone (main ingredient in smog) and particulate matter 2.5<br>Pollution from diesel engines/exhaust<br>Water quality<br>Drinking water contaminants	Industrial risks<br>Air quality<br>Water quality	Environmental
**59**	Maizlish et al. (2019) California Healthy Places Index (HPI) [75]	Family structure/Demographics<br>Percentage of family households with children aged <18 with 2 parents<br>Income and Wealth<br>Percentage of population with household income >200% of the federal poverty level<br>Percentage of population aged 25-64 employed<br>Median household income<br>Education<br>Percentage of population aged >25 with a bachelor's degree or higher<br>Percentage of teenagers aged 15-17 enrolled in school<br>Percentage of children aged 3 or 4 enrolled in preschool<br>Health<br>Health insurance<br>Percentage of adults aged 18-64 currently insured<br>Housing<br>Percentage of housing units occupied by property owners<br>Percentage of households with complete kitchen facilities and plumbing<br>Percentage of low-income home owners paying >50% of income on housing costs<br>Percentage of low-income renter households paying >50% of income on housing costs<br>Percentage of households with ≤1 occupant per room<br>Build environment<br>Percentage of population living within a half-mile of a park, beach, or open space >1 acre<br>Population-weighted percentage of the census tract area with tree canopy	Family structure/ Demographics<br>Income and Wealth<br>Education<br>Health:<br>• Health insurance<br>Housing<br>Build environment<br>Air quality<br>Water quality<br>Social environment<br>Transportation<br>Racial composition	Social, Material, Environmental, Health

Percentage of urban and small-town population residing <1 half-mile from a supermarket or large grocery store

Percentage of rural population living <1 mile from a supermarket or large grocery store

Percentage of population residing within 1-quarter mile of an off-site sales alcohol outlet

Combined employment density for retail, entertainment, and educational uses (jobs per acre)

Air quality

Spatial distribution of gridded diesel particulate-matter emissions from on-road and non-road sources for a summer day in July 2012 (kg/d) (census tracts were ordered by diesel particulate-matter concentration values and assigned a percentile based on the statewide distribution of values)

Mean of summer months (May-October) of the daily maximum 8-h ozone concentration (parts per million), averaged over 3 y (2012-2014) (census tracts were ordered by ozone concentration values and assigned a percentile based on the statewide distribution of values Annual mean concentration of particulate matter ≤2.5 µm across (PM2.5; average of quarterly means, $\mu g/m^3$) over 3y (2012-2014) (census tracts were ordered by PM2.5 concentration values and assigned a percentile based on the statewide distribution of values)

Water quality

Cal EnviroScreen 3.0 drinking water contaminant index for selected contaminants

Social environment

Percentage of registered voters who voted in the 2012 general election

Transportation

Percentage of households with access to an automobile

Percentage of workers (aged ≥16) commuting by walking, cycling, or transit (excluding working from home)

Racial composition

Index of dissimilarity using county percentage of black persons as a parity reference and aggregating census block differences to census tracts

**60**	Acevedo-Garcia et al. (2014) Child Opportunity Index [76]	Education<br>School poverty rate (eligibility for free or reduced-price lunch)<br>Student math proficiency level<br>Student reading proficiency level<br>Proximity to licensed early childhood education centers<br>Proximity to high-quality early childhood education centers<br>Early childhood education participation<br>High school graduation rate<br>Adult educational attainment<br>Housing<br>Housing vacancy rate<br>Foreclosure rate<br>Income and Wealth<br>Poverty rate<br>Public assistance rate<br>Employment/Occupation<br>Unemployment rate<br>Build environment<br>Retail healthy food environment index<br>Proximity to parks and open spaces<br>Proximity to employment<br>Proximity to health care facilities<br>Industrial risks<br>Volume of nearby toxic waste release<br>Proximity to toxic waste release sites	Education<br>Housing<br>Income and Wealth<br>Employment/Occupation<br>Build environment<br>Industrial risks	Social, Material, Environmental

References

1. Zadnik, V.; Reich, B.J. Analysis of the relationship between socioeconomic factors and stomach cancer incidence in Slovenia. *Neoplasma* **2006**, *53*, 103–10.
2. Meijer, M.; Engholm, G.; Grittner, U.; Bloomfield, K. A socioeconomic deprivation index for small areas in Denmark. *Scand. J. Public Health* **2013**, *41*, 560–9. https://doi.org/10.1177/1403494813483937.

3. Benmarhnia, T.; Laurian, L.; Deguen, S. Measuring Spatial Environmental Deprivation: A New Index and its Application in France. *Environmental Justice* **2013**, *6*, 48–55. https://doi.org/10.1089/env.2013.0001.
4. Havard, S.; Deguen, S.; Bodin, J.; Louis, K.; Laurent, O.; Bard, D. A small-area index of socioeconomic deprivation to capture health inequalities in France. *Soc. Sci. Med*. **2008,** *67*, 2007–16. https://doi.org/ 10.1016/j.socscimed.2008.09.031.
5. Rey, G.; Jougla, E.; Fouillet, A.; Hémon, D. Ecological association between a deprivation index and mortality in France over the period 1997 - 2001: variations with spatial scale, degree of urbanicity, age, gender and cause of death. *BMC Public Health* **2009**, *9*, 33. https://doi.org/10.1186/1471-2458-9-33.
6. Lecoffre, C.; Decool, E.; Olié, V. Mortalité cardio-neuro-vasculaire et désavantage social en France en 2011. *Bull. Epidémiol. Hebd.* **2016**, *(20-21)*, 352–8. (In French)
7. Lasbeur, L.; Kaminski, M.; Ancel, P.; Du Mazaubrun, C.; Zeitlin, J. Analyser les inégalités socio-économiques de santé à partir des données du recensement: L'exemple de la grande prématurité à Paris-Petite couronne. *Population* **2006**, *61*, 567–584. https://doi.org/10.3917/popu.604.0567.
8. Lalloué, B.; Monnez, J.M.; Padilla, C.; Kihal, W.; Le Meur, N.; Zmirou-Navier, D.; Deguen, S. A statistical procedure to create a neighborhood socioeconomic index for health inequalities analysis. *Int. J. Equity. Health* **2013**, *12*, 21. https://doi.org/10.1186/1475-9276-12-21.
9. Hagedoorn, P.; Vandenheede, H.; Vanthomme, K.; Willaert, D.; Gadeyne, S. A cohort study into head and neck cancer mortality in Belgium (2001-11): Are individual socioeconomic differences conditional on area deprivation? *Oral Oncol.* **2016**, *61*, 76–82. https://doi.org/10.1016/j.oraloncology.2016.08.014.
10. Splichalova, A.; Slachtova, H.; Tomaskova, H.; Polaufova, P. Analysis of Mortality in the Czech Republic Using the Newly Constructed Socioeconomic Deprivation Index. *Epidemiology* **2007**, 18, S19. https://doi.org/10.1097/01.ede.0000276469.05896.8d.
11. Winkleby, M.; Sundquist, K.; Cubbin, C. Inequities in CHD incidence and case fatality by neighborhood deprivation. *Am. J. Prev. Med.* **2007**, *32*, 97–106.https://doi.org/10.1016/j.amepre.2006.10.002.
12. Ruiz-Ramos, M.; Sánchez, J.; Garrucho, G.; Viciana, F. Inequalities in mortality in Seville. *Gac. Sanit.* **2004**, *18*, 16–23. (In Spanish) https://doi.org/10.1016/s0213-9111(04)71994-9.
13. Salcedo, N.; Saez, M.; Bragulat, B.; Saurina C. Does the effect of gender modify the relationship between deprivation and mortality? *BMC Public Health* 2012, *12*, 574. https://doi.org/ 10.1186/1471-2458-12-574.
14. Domínguez-Berjón, M.F.; Borrell, C.; Cano-Serral, G.; Esnaola, S.; Nolasco, A.; Pasarín, M.I.; Ramis, R.; Saurina, C.; Escolar-Pujolar, A. Constructing a deprivation index based on census data in large Spanish cities (the MEDEA project). *Gac. Sanit.* **2008**, *22*, 179–87. (In Spanish) https://doi.org/10.1157/13123961.
15. Compés Dea, M.L.; Olivan Bellido, E.; Feja Solana, C.; Aguilar Palacio, I.; García-Carpintero Romero Del Hombrebueno, G.; Adiego Sancho, B. Construction of a deprivation index by Basic Healthcare Area in Aragon using Population and Housing Census 2011. *Rev. Esp. Salud. Publica*. **2018**, *92*, e201812087. (In Spanish)

16. Duque, I.; Domínguez-Berjón, M.F.; Cebrecos, A.; Prieto-Salceda, M.D.; Esnaola, S.; Calvo Sánchez, M.; Marí-Dell'Olmo, M. Deprivation index by enumeration district in Spain, 2011. *Gac. Sanit.* **2021**, 35, 113-122. (In Spanish) https://doi.org/ 10.1016/j.gaceta.2019.10.008.
17. Colls C, Mias M, García-Altés A. A deprivation index to reform the financing model of primary care in Catalonia. *Gac. Sanit.* **2020**, *34*, 44–50. (In Spanish) https://doi.org/10.1016/j.gaceta.2018.07.015. (In Spanish)
18. Álvarez-del Arco, D.; Vicente Sánchez, M.; Alejos, B.; Pascual, C.; Regidor, E. Process and results of constructing a deprivation index for the districts of Madrid and Barcelona, Spain. *Rev. Esp. Salud. Publica*. **2013**, *87*, 317–29. (In Spanish) https://doi.org/10.4321/S1135-57272013000400003.
19. Ocaña-Riola, R.; Saurina, C.; Fernández-Ajuria, A.; Lertxundi, A.; Sánchez-Cantalejo, C.; Saez, M.; Ruiz-Ramos, M.; Barceló, M.A.; March, J.C.; Martínez, J.M.; Daponte, A.; Benach, J. Area deprivation and mortality in the provincial capital cities of Andalusia and Catalonia (Spain). *J. Epidemiol. Community Health* **2008**, *62*, 147–52. https://doi.org/10.1136/jech.2006.053280.
20. Benach, J.; Yasui, Y. Geographical patterns of excess mortality in Spain explained by two indices of deprivation. *J. Epidemiol. Community Health* **1999**, *53*, 423–31. https://doi.org/10.1136/jech.53.7.423
21. Sánchez-Cantalejo, C.; Ocana-Riola, R.; Fernández-Ajuria, A. Deprivation index for small areas in Spain. *Soc. Indic. Res.* **2008**, *89*, 259–273. https://doi.org/10.1007/s11205-007-9114-6.
22. García-Gil, C.; Cruz-Rojo, C.; Alvarez-Girón, M.; Solano-Parés, A. Health inequalities in Seville, Spain: use of indicators of social deprivation and mortality in small areas. *Public Health.* **2004,** *118*, 11–20. https://doi.org/ 10.1016/S0033-3506(03)00141-0.
23. Esnaola, S.; Aldasoro, E.; Ruiz, R.; Audicana, C.; Pérez, Y.; Calvo, M. Socioeconomic inequalities in mortality in the Basque Country. *Gac. Sanit.* **2006**, *20*, 16–24. (In Spanish) https://doi.org/ 10.1157/13084123.
24. Panczak, R.; Galobardes, B.; Voorpostel, M.; Spoerri, A.; Zwahlen, M.; Egger, M.; Swiss National Cohort and Swiss Household Panel. A Swiss neighbourhood index of socioeconomic position: development and association with mortality. *J. Epidemiol. Community Health* **2012**, *66*, 1129–36. https://doi.org/ 10.1136/jech-2011-200699.
25. Juhász, A.; Nagy, C.; Páldy, A.; Beale, L. Development of a Deprivation Index and its relation to premature mortality due to diseases of the circulatory system in Hungary, 1998-2004. *Soc. Sci. Med.* **2010**, *70*, 1342–9. https://doi.org/10.1016/j.socscimed.2010.01.024.
26. Caranci, N.; Biggeri, A.; Grisotto, L.; Pacelli, B.; Spadea, T.; Costa, G. The Italian deprivation index at census block level: definition, description and association with general mortality. *Epidemiol. Prev*. **2010**, *34*, 167–76. (In Italian)
27. Rosano, A.; Pacelli, B.; Zengarini, N.; Costa, G.; Cislaghi, C.; Caranci, N. Update and review of the 2011 Italian deprivation index calculated at the census section level. *Epidemiol. Prev*. **2020**, *44*, 162–170. (In Italian) https://doi.org/ 10.19191/EP20.2-3.P162.039.
28. Ivaldi, E.;Testi, A. Genoa index of deprivation (GDI): An index of material deprivation for geographical areas. In C. M. Baird (Ed.), Social indicators: Statistics, trends and policy development. Hauppauge, New York: Nova Science Publishers. 2011.
29. Cadum, E.; Costa, G.; Biggeri, A.; Martuzzi, M. Deprivation and mortality: a deprivation index suitable for geographical analysis of inequalities. *Epidemiol. Prev.* **1999**, *23*, 175–87. (In Italian)

30. Michelozzi, P.; Perucci, C.A.; Forastiere, F.; Fusco, D.; Ancona, C.; Dell'Orco, V. Inequality in health: socioeconomic differentials in mortality in Rome, 1990-95. *J. Epidemiol. Community Health* **1999**, *53*, 687–93. https://doi.org/10.1136/jech.53.11.687.
31. Petrelli, A.; Gnavi, R.; Marinacci, C.; Costa, G. Socioeconomic inequalities in coronary heart disease in Italy: a multilevel population-based study. *Soc. Sci. Med.* **2006**, *63*, 446–56. https://doi.org/10.1016/j.socscimed.2006.01.018.
32. Jordan, H.; Roderick, P.; Martin, D. The Index of Multiple Deprivation 2000 and accessibility effects on health. *J. Epidemiol. Community Health* **2004**, *58*, 250–7. https://doi.org/10.1136/jech.2003.013011.
33. English indices of deprivation 2019: technical report
34. Townsend, P.; Phillimore, P.; Beattie, A. Health and Deprivation: Inequality and The North. Croom Helm. 1988.
35. Jarman, B. Identification of underprivileged areas. *Br. Med. J. (Clin. Res. Ed.)* **1983**, *286*, 1705–9. https://doi.org/10.1136/bmj.286.6379.1705.
36. Carstairs, V.; Morris, R. Deprivation: explaining differences in mortality between Scotland and England and Wales. *Br. Med. J.* **1989**, *299*, 886–9. https://doi.org/10.1136/bmj.299.6704.886.
37. Simpson, L. The Department of Environment's index of local conditions: Don't touch it. *Radical Statistics* **1995**, *61*, 13–25.
38. Department of the Environment, Transport and the Regions. 1998 Index of Local Deprivation: A Summary of Results. London: DETR. 1998. Available online: https://www.legco.gov.hk/yr99-00/english/bc/bc09/papers/1471e01.pdf (accessed 26 October 2021).
39. Richardson, E.A.; Mitchell, R.; Shortt, N.K.; Pearce, J.; Dawson, T.P. Developing Summary Measures of Health-Related Multiple Physical Environmental Deprivation for Epidemiological Research. *Environment and Planning A: Economy and Space* **2010**, *42*, 1650–1668. https://doi.org/10.1068/a42459.
40. Noble, M.; Lloyd, M.; Wright, G.; Dibben, C.; Smith, G. Developing deprivation measures for Northern Ireland. *Journal of the Statistical and Social Inquiry Society of Ireland* **2001/2002**, *XXXI*, 5–25.
41. IJpelaar, J.; Power, T.; Green, B. Northern Ireland Multiple Deprivation Measures 2017. *Journal of Statistical and Social Inquiry Society of Ireland* **2018-19**, *48*, 163-174.
42. Kelly, A.; Sinclair, H. Deprivation and health: identifying the black spots. *Journal of Health Gain* **1997**, 1, 13–14.
43. Teljeur, C.; Darker, C.D.; Barry, J.; O'Dowd, T. The Trinity national deprivation index for health and health services research 2016. Department of Public Health & Primary Care. Trinity College Dublin. 2019. Available online: https://www.tcd.ie/medicine/public_health_primary_care/assets/pdf/Trinity-deprivation-report-11-2019.pdf (accessed 26 October 2021).
44. Haase, T.; Pratschke, J.; Gleeson, J. All-Island Deprivation Index: Towards the development of consistent deprivation measures for the Island of Ireland. *Borderlands: The Journal of Spatial Planning in Ireland* **2012**, *2,* 21–37.
45. University of Oxford—Department of Social Policy and Social Work. Index of Multiple Deprivation for Wales. Available online: http://gov.wales/docs/statistics/2011/110831wimd2000en.pdf (accessed 26 October 2021).
46. Welsh Index of Multiple Deprivation (full Index update with ranks): 2019 | GOV.WALES. Available online: https://gov.wales/welsh-index-multiple-deprivation-full-index-update-ranks-2019 (accessed 26 October 2021).

47. Scottish index of multiple deprivation 2004: summary technical report. Available online: https://www.webarchive.org.uk/wayback/archive/20200207120514/https://www2.gov.scot/Topics/Statistics/SIMD/Publications (accessed 26 October 2021).
48. Scottish index of Multiple Deprivation (full Index update with ranks): 2020 | GOV.SCOT. Available online: https://www.gov.scot/collections/scottish-index-of-multiple-deprivation-2020/ (accessed 26 October 2021).
49. Salmond, C.E.; Crampton, P. Development of New Zealand's deprivation index (NZDep) and its uptake as a national policy tool. *Can J. Public Health* **2012**, *103*, S7–11.
50. Socioeconomic Deprivation Indexes: NZDep and NZiDep, Department of Public Health (full Index update). Available online: https://www.otago.ac.nz/wellington/departments/publichealth/research/hirp/otago020194.html (accessed 26 October 2021).
51. Trewin, D. Socio-economic indexes for areas Australia. Belconnen ACT: Australian Bureau of Statistics. 2003.
52. Socio-Economic Indexes for Areas (full Index update) Available online: https://www.abs.gov.au/websitedbs/censushome.nsf/home/seifa (accessed 26 October 2021).
53. Bell, N.; Schuurman, N.; Oliver, L.; Hayes, V.M. Towards the construction of place specific measures of deprivation: A case study from the Vancouver Metropolitan area. *Canadian Geographer* **2007**, *51*, 444–61 https://doi.org/10.1111/j.1541-0064.2007.00191.x.
54. Langlois, A.; Kitchen, P. Identifying and Measuring Dimensions of Urban Deprivation in Montreal: An Analysis of the 1996 Census Data. *Urban Studies* **2001**, *38*, 119–139. https://doi.org/10.1080/00420980020014848.
55. Pampalon, R.; Raymond, G. A deprivation index for health and welfare planning in Quebec. *Chronic. Dis. Can.* **2000**, *21*, 104–13.
56. Pampalon, R.; Hamel, D.; Gamache, P.; Simpson, A.; Philibert, M.D. Validation of a deprivation index for public health: a complex exercise illustrated by the Quebec index. *Chronic. Dis. Inj. Can*. **2014**, *34*, 12–22.
57. Mustard, C.A.; Frohlich, N. Socioeconomic status and the health of the population. *Med. Care* **1995**, 33, DS43–54. https://doi.org/10.1097/00005650-199512001-00007.
58. Metge, C.; Chateau, D.; Prior, H.; Soodeen, R.; De Coster, C.; Barre, L. Composite Measures/Indices of Health and Health System Performance. Winnipeg, MB: Manitoba Centre for Health Policy. 2009.
59. Bell, N.; Schuurman, N.; Hayes, M.V. Using GIS-based methods of multicriteria analysis to construct socio-economic deprivation indices. *Int. J. Health Geogr.* **2007**, *6,* 17. https://doi.org/10.1186/1476-072X-6-17.
60. Butler, D.C.; Petterson, S.; Phillips, R.L.; Bazemore, A.W. Measures of social deprivation that predict health care access and need within a rational area of primary care service delivery. *Health Serv. Res*. **2013**, *48*, 539–59. https://doi.org/ 10.1111/j.1475-6773.2012.01449.x.
61. Andrews, M.R.; Tamura, K.; Claudel, S.E.; Xu, S.; Ceasar, J.N.; Collins, B.S.; Langerman, S.; Mitchell, V.M.; Baumer, Y.; Powell-Wiley, T.M. Geospatial Analysis of Neighborhood Deprivation Index (NDI) for the United States by County. *J. Maps* **2020**, *16*, 101–112. https://doi.org/10.1080/17445647.2020.1750066.
62. Singh, G.K.; Miller, B.A.; Hankey, B.F.; Feuer, E.J.; Pickle, L.W. Changing area socioeconomic patterns in U.S. cancer mortality, 1950-1998: Part I--All cancers among men. *J. Natl. Cancer Inst.* **2002**, 94, 904–15. https://doi.org/ 10.1093/jnci/94.12.904.

63. Kind, A.J.H.; Buckingham, W. Making Neighborhood Disadvantage Metrics Accessible: The Neighborhood Atlas*. New England Journal of Medicine* **2018**, *378*, 2456–2458. https://doi.org/10.1056/NEJMp1802313.
64. Diez-Roux, A.V.; Kiefe, C.I.; Jacobs, D.R. Jr.; Haan, M.; Jackson, S.A.; Nieto, F.J.; Paton, C.C.; Schulz, R. Area characteristics and individual-level socioeconomic position indicators in three population-based epidemiologic studies**.** *Ann. Epidemiol*. **2001**, *11*, 395–405. https://doi.org/ 10.1016/s1047-2797(01)00221-6.
65. Sampson, R.J. Neighborhoods and Violent Crime: A Multilevel Study of Collective Efficacy*. Science* **1997**, *277*, 918–924. https://doi.org/10.1126/science.277.5328.918.
66. Perkins, P.; Royster, M.; Studer, K.; Wibberly, K.; Reed, K.; Henry, K.; Anson-Dwamena, T. Developing Healthy Communities: The Health Opportunity Index. American Public Health Association (APHA) 140th Annual Meeting, San Francisco, California, USA, 27–31 October 2012.
67. Messer, L.C.; Laraia, B.A.; Kaufman, J.S.; Eyster, J.; Holzman, C.; Culhane, J.; Elo, I.; Burke, J.G.; O'Campo, P. The development of a standardized neighborhood deprivation index. *J. Urban Health* **2006**, *83*, 1041–62. https://doi.org/ 10.1007/s11524-006-9094-x.
68. Lòpez-De Fede, A.; Stewart, J.E.; Hardin, J.W.; Mayfield-Smith, K. Comparison of small-area deprivation measures as predictors of chronic disease burden in a low-income population. *Int. J. Equity Health* **2016**, *15*, 89. https://doi.org/ 10.1186/s12939-016-0378-9.
69. Brokamp, C.; LeMasters, G.K.; Ryan, P.H. Residential mobility impacts exposure assessment and community socioeconomic characteristics in longitudinal epidemiology studies. *J. Expo. Sci. Environ. Epidemiol.* **2016**, *26*, 428–34. https://doi.org/10.1038/jes.2016.10.
70. Brokamp, C.; Beck, A.F.; Goyal, N.K.; Ryan, P.; Greenberg, J.M.; Hall, E.S. Material community deprivation and hospital utilization during the first year of life: an urban population-based cohort study. *Ann. Epidemiol*. **2019**, *30*, 37–43. https://doi.org/10.1016/j.annepidem.2018.11.008.
71. Wheeler, D.C.; Raman, S.; Jones, R.M.; Schootman, M.; Nelson, E.J. Bayesian deprivation index models for explaining variation in elevated blood lead levels among children in Maryland. *Spat. Spatiotemporal Epidemiol*. **2019**, *30*, 100286. https://doi.org/ 10.1016/j.sste.2019.100286.
72. Kolak, M.; Bhatt, J.; Park, Y.H.; Padrón, N.A.; Molefe, A. Quantification of Neighborhood-Level Social Determinants of Health in the Continental United States. *JAMA Netw. Open*. **2020**, *3*, e1919928. https://doi.org/ 10.1001/jamanetworkopen.2019.19928.
73. Dubowitz,T.; Heron, M.; Bird, C.E.; Lurie, N.; Finch, B.K.; Basurto-Dávila, R.; Hale, L.; Escarce, J.J. Neighborhood socioeconomic status and fruit and vegetable intake among whites, blacks, and Mexican Americans in the United States. *Am. J. Clin. Nutr*. **2008**, *87*, 1883–91. https://doi.org/ 10.1093/ajcn/87.6.1883.
74. Peyvandi, S.; Baer, R.J.; Chambers, C.D.; Norton, M.E.; Rajagopal, S.; Ryckman, K.K.; Moon-Grady, A.; Jelliffe-Pawlowski, L.L.; Steurer, M.A. Environmental and Socioeconomic Factors Influence the Live-Born Incidence of Congenital Heart Disease: A Population-Based Study in California. *J. Am. Heart. Assoc.* **2020**, *9*, e015255. https://doi.org/10.1161/JAHA.119.015255.
75. Maizlish, N.; Delaney, T.; Dowling, H.; Chapman, D.A.; Sabo, R.; Woolf, S.; Orndahl, C.; Hill, L.; Snellings, L. California Healthy Places Index: Frames Matter. *Public Health Rep.* **2019**, *134*, 354-362. https://doi.org/10.1177/0033354919849882.
76. Acevedo-Garcia, D.; McArdle, N.; Hardy, E.F.; Crisan, U.I.; Romano, B.; Norris, D.; Baek, M.; Reece, J. The child opportunity index: improving collaboration between community development and public health. *Health Aff. (Millwood)* **2014**, *33*, 1948–57. https://doi.org/10.1377/hlthaff.2014.0679.
